# Single-cell RNA sequencing and immune microenvironment analysis reveal PLOD2-driven malignant transformation in cervical cancer

**DOI:** 10.3389/fimmu.2024.1522655

**Published:** 2025-01-07

**Authors:** Zhiheng Lin, Fengxin Wang, Renwu Yin, Shengnan Li, Yuquan Bai, Baofang Zhang, Chenlin Sui, Hengjie Cao, Dune Su, Lianwei Xu, Honghong Wang

**Affiliations:** ^1^ Department of Gynecology, Longhua Hospital, Shanghai University of Traditional Chinese Medicine, Shanghai, China; ^2^ The Third Affiliated Hospital of Beijing University of Chinese Medicine, Beijing University of Chinese Medicine, Beijing, China; ^3^ Department of Urology, Longhua Hospital Shanghai University of Traditional Chinese Medicine, Shanghai, China

**Keywords:** cervical cancer, single-cell RNA sequencing, tumor microenvironment, prognostic model, immune evasion, therapeutic targets

## Abstract

**Background:**

Cervical cancer is the fourth most common cancer in women globally, and the main cause of the disease has been found to be ongoing HPV infection. Cervical cancer remains the primary cause of cancer-related death despite major improvements in screening and treatment approaches, especially in low- and middle-income nations. Therefore, it is crucial to investigate the tumor microenvironment in advanced cervical cancer in order to identify possible treatment targets.

**Materials and methods:**

In order to better understand malignant cervical cancer epithelial cells (EPCs), this study used bulk RNA-seq data from UCSC in conjunction with single-cell RNA sequencing data from the ArrayExpress database. After putting quality control procedures into place, cell type identification and clustering analysis using the Seurat software were carried out. To clarify functional pathways, enrichment analysis and differential gene expression were carried out. The CIBERSORT and ESTIMATE R packages were used to evaluate the immune microenvironment characteristics, and univariate and multivariate Cox regression analyses were used to extract prognostic features. Furthermore, assessments of drug sensitivity and functional enrichment were carried out.

**Results:**

Eight cell types were identified, with EPCs showing high proliferative and stemness features. Five EPC subpopulations were defined, with C1 NNMT+ CAEPCs driving tumor differentiation. A NNMT CAEPCs Risk Score (NCRS) model was developed, revealing a correlation between elevated NCRS scores and adverse patient outcomes characterized by immune evasion. *In vitro* experiments validated that the prognostic gene PLOD2 significantly enhances proliferation, migration, and invasion of cervical cancer cells.

**Conclusion:**

This investigation delineated eight cell types and five subpopulations of malignant EPCs in cervical cancer, establishing the C1 NNMT+ CAEPCs as a crucial therapeutic target. The NCRS model demonstrated its prognostic capability, indicating that higher scores are associated with poorer clinical outcomes. The validation of PLOD2 as a prognostic gene highlights its therapeutic potential, underscoring the critical need for integrating immunotherapy and targeted treatment strategies to enhance diagnostic and therapeutic approaches in cervical cancer.

## Introduction

Cervical cancer ranks as the fourth most prevalent malignancy among women globally, with incidence and mortality rates of 13.1% and 6.1%, respectively (1, 2). It continues to be the primary cause of cancer-related mortality in nations with few resources (3), primarily due to persistent infection with HPV. Although significant reductions in cervical cancer rates have been achieved in certain developed regions through HPV vaccination, effective screening programs, and advancements in diagnostic and adjuvant therapies (4), a study from 2020 revealed that nearly 90% of cervical cancer fatalities occur in low- and middle-income countries (5). Current treatment modalities encompass conventional chemotherapy regimens utilizing cisplatin and paclitaxel, radiotherapy (6), targeted therapies like bevacizumab, and immunotherapies employing immune checkpoint inhibitors (7). The dearth of treatment choices for cervical cancer that is advanced, recurring, or metastatic emphasizes the critical need for additional research into the tumor microenvironment in order to identify novel therapeutic targets.

An increasing number of studies have highlighted the critical role of the tumor microenvironment (TME) in understanding tumor progression and drug resistance (8). Exploring the TME in different types of tumors holds significant clinical value (9–11). Techniques such as high-throughput sequencing and single-cell RNA sequencing (scRNA-seq) have enabled researchers to deeply analyze cellular heterogeneity, immune evasion mechanisms, and the tumor’s response to therapy within the TME (12). ScRNA-seq has transformed our understanding of tumor heterogeneity, particularly enhancing the exploration of the TME (13). It has also significantly advanced our understanding of the mechanisms and evolutionary pathways underlying tumor development (14), For example, single-cell sequencing analysis has been widely used in breast cancer research to analyze intercellular communication within the TME and explore key factors that influence tumor heterogeneity (15–18), scRNA-seq has been widely employed to investigate cervical cancer heterogeneity (19, 20). Our research focuses on malignant epithelial cells in cervical cancer, as chronic HPV infection primarily targets the squamous epithelium, and HPV’s life cycle is intricately tied to the differentiation of host epithelial cells (21).

This study sought to explore the variability of particular cell types in order to clarify the reasons behind the advancement of cervical cancer. Using scRNA-seq, we performed a thorough investigation of malignant cervical cancer EPCs and created a unique predictive model to find possible treatment targets. To confirm our findings, we also looked at the tumor immune microenvironment and ran cellular tests. Our study offers new perspectives on potential cervical cancer treatment approaches.

## Methods

### Acquisition of cervical cancer scRNA-seq data

The ArrayExpress database provided the scRNA-seq data for cervical cancer used in this investigation (accession number E-MTAB-12305) (22). Additionally, bulk RNA-seq data, which included information on somatic mutations and clinical variables (such as age, race, tumor stage, and survival time), were retrieved from the University of California, Santa Cruz (UCSC, https://xena.ucsc.edu/).

### ScRNA-seq data quality control and cell type identification

The “Seurat” R package (version 4.3.0) was used to transform the scRNA-seq data into Seurat objects (23). To identify and exclude doublet cells, the “DoubletFinder” R package (version 2.0.3) (24) was applied. Subsequent filtering was conducted based on specific criteria: features between 300 and 6,000 (nFeature), counts ranging from 500 to 50,000 (nCount), and fewer than 5% of genes associated with red blood cells. Additionally, cells with mitochondrial gene expression levels higher than 25% of the total were not included in the analysis.

The filtered cell data was then normalized using the “NormalizeData” function, and the “FindVariableFeatures” function (25–27) was utilized to identify the top 2,000 highly variable genes. The “ScaleData” function was then used to normalize the data (28, 29). The “CellCycleScoring” function was utilized to evaluate cell cycle phases, followed by dimensionality reduction via “RunPCA,” where the first 30 principal components (PCs) were selected for subsequent analysis. To mitigate batch effects across samples, the “harmony” R package (v0.1.1) was implemented (30, 31). The “FindNeighbors” and “FindClusters” projects were used to cluster cells, and the annotation of the clusters was done using previously identified marker genes. Visualization of the clusters was performed using Uniform Manifold Approximation and Projection (UMAP) (32, 33).

### InferCNV analysis

To differentiate tumor cells from non-tumor cells, we utilized single-cell sequencing data and chromosomal sorting, employing the CNV algorithm via the “InferCNV” R package (34). EPCs exhibiting heightened copy number variations were classified as Cancer-Associated EPCs (CAEPCs). Additionally, we conducted InferCNV analysis on the identified tumor subpopulations.

### Identification and enrichment of DEGs in CAEPC subpopulations

Differentially expressed genes (DEGs) within CAEPC subpopulations were identified using the “FindAllMarkers” function from the Seurat package. We further employed the Ro/e algorithm to evaluate tissue-specific expression patterns and cell cycle preferences within these subpopulations. Subsequently, the identified DEGs underwent Gene Ontology Biological Process (GOBP) enrichment analysis utilizing the “ClusterProfiler” projects. We used data from the Molecular Signature Database (MSigDB) to perform Gene Set Enrichment Analysis (GSEA) in order to identify significantly enriched pathways that reflect collective gene expression trends.

### Single-cell pseudotime analysis

We explored the potential differentiation states and developmental trajectories of CAEPC subpopulations in cervical cancer through comparative analyses using CytoTRACE, Monocle 2, and Slingshot methods. The CytoTRACE analysis (35) predicted the stemness and relative differentiation states of CAEPC subpopulations using scRNA-seq data, facilitating the identification of developmental trajectories for CAEPC cells (36). Employing Monocle 2 (version 2.24.0), we mapped CAEPC subpopulations along pseudotime trajectories, inferred their single-cell developmental paths, and generated UMAP plots for dimensionality reduction (37). To infer cell lineages and developmental trajectories from gene expression data, we utilized the “Slingshot” R package (v2.6.0) (38), with the “getlineage” function specifically identifying cell lineages and tracking trajectories across subpopulations.

### Transcription factor and cell interaction analysis

To investigate gene regulatory networks in cervical cancer subgroups, we applied scRNA-seq data in conjunction with the pySCENIC package (version 0.10.0) to analyze transcription factor (TF) enrichment and regulator activity. Using the AUCell tool, we scored regulator activities, selecting the top five TFs in each subgroup based on highest scores and examined their expression patterns across subgroups.

In addition, we used the “cellchat” package (version 1.6.1) (39, 40) to measure and visualize the intensity of incoming and outgoing signaling across various cervical cancer cell types. In order to determine the frequency and strength of intercellular contacts, we also examined the levels of receptor-ligand expression among important subgroups.

### Development of prognostic features and nomogram construction

With the purpose of predicting overall survival, we have identified and validated predictive characteristics using bulk RNA-seq data from patients with cervical cancer. Using the top 100 highly expressed genes from the CAEPC subpopulations, univariate Cox regression analysis was used to identify genes significantly linked with the prognosis of cervical cancer (P < 0.05). We refined the gene set most closely associated with prognosis by applying Least Absolute Shrinkage and Selection Operator (LASSO) analysis (41, 42) to determine the optimal λ and prevent overfitting. The coefficients for each prognostic gene were then found using multivariate Cox regression analysis, and risk scores were then computed using the following formula: risk score == $\sum_{\text{i}}^{\text{n}}\text{Xi}\times \text{Yi}$ (where Y signifies the degree of gene expression and X the coefficient. Kaplan-Meier survival curves were generated using the “Survival” R package (version 3.3.1) to compare survival outcomes across groups.

To enhance the nomogram’s predictive capacity, we integrated clinicopathological factors—including age, race, tumor stage, and survival time—into a multivariate Cox regression analysis. Using the “rms” R package, we constructed a nomogram to estimate 1-year, 3-year, and 5-year overall survival (OS) rates for cervical cancer patients. The model’s performance was subsequently assessed through receiver operating characteristic (ROC) curve analysis (43–45).

### Immune microenvironment analysis

We analyzed the immune microenvironment utilizing the “CIBERSORT” R package (version 0.1.0) (46) to quantify the proportions of various immune cell types present in cervical cancer. Correlation analyses among immune cell types, prognosis-associated genes, and risk scores were conducted using the “corrplot” R package (version 0.92). Additionally, the Immune Score, Stromal Score, ESTIMATE Score, and tumor purity in patients with cervical cancer were evaluated using the “ESTIMATE” R package (version 1.0.13) (47, 48). In addition, we looked at Tumor Immune Dysfunction and Exclusion (TIDE) scores from various patient groups.

### Mutation analysis

The “maftools” R package (49) was utilized to examine somatic mutation data for patients with cervical cancer, hence enabling our investigation of tumor mutation burden (TMB) (50). To assess the link between risk scores and TMB, we used a Spearman correlation analysis.

### Functional enrichment and GSVA analysis

The “DESeq2” R program was used to identify DEGs. The Kyoto Encyclopedia of Genes and Genomes (KEGG) (51–53) and Gene Ontology (GO) databases (54) were then used for enrichment analyses, with an emphasis on molecular function (MF), cellular component (CC), and biological process (BP). We used analyses of Hallmark gene sets (55) and the “GSVA” R package to further explore biological states between groups. A statistical significance threshold of 0.05 was used for the P-value.

### Drug sensitivity analysis

Drug sensitivity data were obtained from The Genomics of Drug Sensitivity in Cancer (GDSC) database. The “pRRophetic” R package (version 0.5) (56) was utilized to predict the sensitivity of various chemotherapy drugs for different patient groups, based on the 50% inhibitory concentration (IC50).

### Cell culture

The American Type Culture Collection (ATCC) provided the SiHa and HeLa cell lines used to treat cervical cancer. Under standard conditions of 37°C, 5% CO2 and 95% humidity, both cell lines were kept in a culture medium supplemented with non-essential amino acids, 10% fetal bovine serum (FBS) and 1% penicillin/streptomycin.

### Cell transfection

GenePharma (Suzhou, China) provided the siRNA constructs used for PLOD2 knockdown. The study incorporated a negative control group (si-NC) alongside two PLOD2 knockdown groups (si-PLOD2-1 and si-PLOD2-2). The Lipofectamine 3000 RNAiMAX (Invitrogen, USA) manufacturer’s instructions were followed for transfection.

### CCK-8 assay for cell viability

A CCK-8 test was used to evaluate the viability of the transfected SiHa and HeLa cell lines. Cell suspensions were plated in 96-well plates at a density of 5 × 10³ cells per well and incubated for 24 hours. Following this, 10 μL of CCK-8 reagent (A311-01, Vazyme) was added to each well, and the cells were incubated for an additional 2 hours at 37°C in the dark. On days 1, 2, 3, and 4, cell viability was assessed by measuring absorbance at 450 nm using a Thermo microplate reader (A33978). Optical density (OD) values were visually represented in line graphs.

### Plate cloning assay

SiHa and HeLa cell lines were plated at a density of 1 × 10³ cells per well in 6-well plates. After a 2-week incubation, cells were washed with PBS, fixed with 4% paraformaldehyde, stained with 0.1% Crystal Violet, and photographed for subsequent colony count analysis.

### Cell migration and invasion assay

Transwell assays were conducted to evaluate cell migration and invasion. Cell suspensions were cultured in 24-well plates with chambers that were either coated with Matrigel (BD Biosciences, USA) or left uncoated. The lower chamber contained medium supplemented with 10% FBS. After a 36-hour incubation period, cells in the lower chamber were fixed with 4% paraformaldehyde and stained with 0.1% Crystal Violet. Cell counts were performed using a light microscope.

### Scratch assay

A wound healing assay was performed 48 hours post-transfection. In 6-well plates, transfected cells were cultivated until 95% confluence was achieved. Using a sterile 200 μL pipette tip, a linear scratch was made on the cell monolayer. The media was changed and the cells were incubated some more after a mild PBS wash. The same region was photographed at 0 and 48 hours, and the scratch’s breadth was determined.

### EDU staining

Transfected SiHa and HeLa cells were seeded in 6-well plates at a density of 5 × 10³ cells and incubated for 24 hours at room temperature. An EdU solution was then added to the culture medium and incubated at 37°C for 2 hours. After washing with PBS, the cells were fixed with 4% paraformaldehyde. The cells were then exposed to 0.5% Triton X-100 and 2 mg/mL glycine for 15 minutes. At last, the cells were incubated for half an hour at room temperature using 1 milliliter of Apollo and 1 milliliter of Hoechst staining solution. Cell proliferation was examined using fluorescence microscopy.

### Statistical analysis

R software (version 4.3.0) was used to conduct statistical analysis. Unless otherwise noted, a P-value of less than 0.05 was considered statistically significant.

## Results

### Acquisition of cervical cancer single-cell data and distribution of major clusters

The scRNA-seq data for cervical cancer were sourced from the Array Express database, focusing on samples from two distinct tissue types: tumor (T) and high-grade squamous intraepithelial lesion (HSIL). By applying unsupervised clustering to the top 2000 highly variable genes, we successfully identified a range of cell types, the results of which were visualized in UMAP plots. We categorized cervical cancer cells into eight distinct types: epithelial cells (EPCs), endothelial cells (ECs), myeloid cells, Mast Cells (MCs), B plasma cells, T NK cells, fibroblasts, and neutrophils (**Supplementary Figure S1A**). These cells were derived from six sample sources: H1, H2, T1, T2, T3, and T4, where “H” denoted HSIL and “T” signified tumor tissue, as illustrated in the lower left of **Supplementary Figure S1A**. Notably, T2 served as the primary source of ECs, while T1 and T3 were the main sources of EPCs. In the lower right corner of **Supplementary Figure S1A**, we assessed the cell cycle phases (G1, G2M, and S) for each cell type and found a significant proportion of EPCs in the G2M phase. This suggests that these EPCs are in a high-proliferative state, which could impact tumor progression. The graphs in the top left and upper right of **Supplementary Figure S1A** display the distributions of the Cell Stemness AUC and G2M Score for each cell type, respectively, indicating that certain EPCs exhibited comparatively high G2M Scores. As shown in **Supplementary Figure S1B**, all identified cells originated from HSIL and tumor tissues. The bar chart in **Supplementary Figure S1C** illustrates that the majority of ECs were derived from patient sample T2, with additional contributions from H2, T1, and T4. **Supplementary Figure S1D** highlighted the top five marker genes associated with each cell type across the various tissue sources.

In the subsequent phase of our research, we delved deeper into the EPCs subgroups. We conducted InferCNV analysis to identify malignant cells, categorizing epithelial cells with high Copy Number Variation as CAEPCs (Cancer-Associated EPCs) (**Supplementary Figure S2A**).

**Figure 1:**
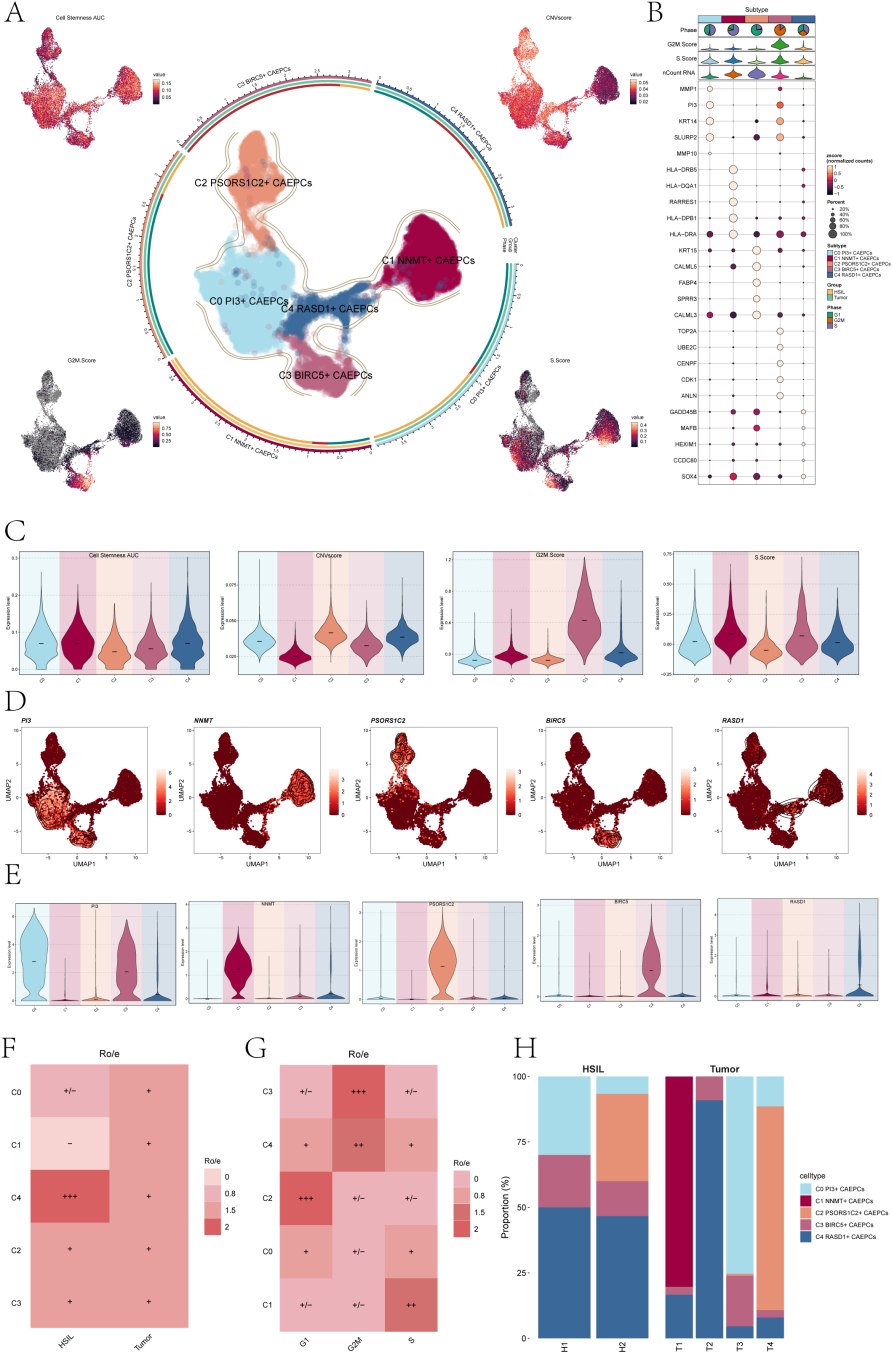
Overview of cervical cancer subpopulations. **(A)** The central UMAP plot demonstrated the distribution of five malignant epithelial cells subpopulations in cervical cancer, while the surrounding UMAP plots illustrated the variations in Cell Stemness AUC, CNV score, G2M Score, and S Score. **(B)** The bubble chart highlighted the top five marker genes associated with each subpopulation. **(C)** The Cell Stemness AUC, CNV score, G2M score, and S score with regard to cervical cancer subpopulation were displayed as violin plots. **(D)** UMAP plots depicted the distribution of signature genes (PI3, NNMT, PSORS1C2, BIRC5, RASD1) across the five subpopulations. **(E)** The expression levels of signature genes within each subpopulation were presented in violin plots. **(F)** A heatmap displayed the Ro/e values reflecting tissue type preferences (HSIL, Tumor) for each cervical cancer subpopulation. (HSIL-High Grade Squamous Intraepithelial Lesion). **(G)** The heatmap also illustrated the Ro/e values related to cell cycle preferences (G1, G2M, S) for each subpopulation. **(H)** A bar chart indicated the specific proportions of cervical cancer subpopulations originating from each tissue type (H1, H2, T1, T2, T3, T4). +++ indicates the highest Ro/e value; ++ indicates the second highest Ro/e value; + indicates a moderate Ro/e value; +/- indicates a lower Ro/e value; - indicates the lowest Ro/e value.

Following the identification of CAEPCs, we carried out unbiased clustering, which, according to their marker genes, identified five different subpopulations: C0 PI3+ CAEPCs, C1 NNMT+ CAEPCs, C2 PSORS1C2+ CAEPCs, C3 BIRC5+ CAEPCs, and C4 RASD1+ CAEPCs. **Figure 1A** illustrated the distribution of these cervical cancer CAEPC subpopulations. For each CAEPC subpopulation, we calculated the Cell Stemness AUC, CNV Score, G2M Score, and S Score, with their distributions visualized in UMAP plots (**Figure 1A**). The top five marker genes for each subpopulation were listed in **Figure 1B**: C0 PI3+ CAEPCs (MMP1, PI3, KRT14, SLURP2, MMP10), C1 NNMT+ CAEPCs (HLA-DRB5,HLA-DQA1,RARRES1,HLA-DPB1, HLA-DRA),C2 PSORS1C2+ CAEPCs (KRT15, CALML5, FABP4, SPRR3, CALML3), C3 BIRC5+ CAEPCs (TOP2A,UBE2C, CENPF, CDK1, ANLN),and C4 RASD1+ CAEPCs (GADD45B,MAFB,HEXIM1,CCDC80,SOX4).

As seen in **Figure 1C**, the Cell Stemness AUC, CNV Score, G2M Score, and S Score were calculated for every group to assess heterogeneity and gauge the stemness and differentiation potential within every CAEPC subpopulation. The results of the analysis showed that the subpopulations of C0 PI3+ CAEPCs, C1 NNMT+ CAEPCs, and C4 RASD1+ CAEPCs had higher Cell Stemness AUC values. Notably, among all subpopulations, the C1 NNMT+ CAEPCs demonstrated the lowest CNV Score relative to the others.

We then investigated the heterogeneity among the various cervical cancer CAEPC subpopulations, using ECs as a reference to infer CNV states via inferCNV (**Supplementary Figure S2B**). The expression of key genes associated with each subpopulation (PI3, NNMT, PSORS1C2, BIRC5, RASD1) was assessed, with their distribution and expression levels depicted in **Figures 1D, E**. The analysis revealed that the PI3 gene of the C0 subpopulation was also expressed in the C3 subpopulation.

We calculated the Ro/e values for different tissue types to understand the tissue preferences of each subpopulation (**Figure 1F**). The data indicated that the C1 subpopulation had the lowest affinity for HSIL tissues, whereas the C4 subpopulation showed a stronger preference for them. Additionally, analysis of cell cycle preferences across the subpopulations (**Figure 1G**) revealed that the C2 subpopulation favored the G1 phase, while the C3 subpopulation preferred the G2M phase. Moreover, the tissue origins of the five cervical cancer CAEPCs subpopulations were examined, showing that C2 PSORS1C2+ CAEPCs primarily originated from H2 and T4, whereas C1 NNMT+ CAEPCs mainly derived from T1 (**Figure 1H**).

### Enrichment analysis of cervical cancer CAEPCs subpopulations

**Figure 2:**
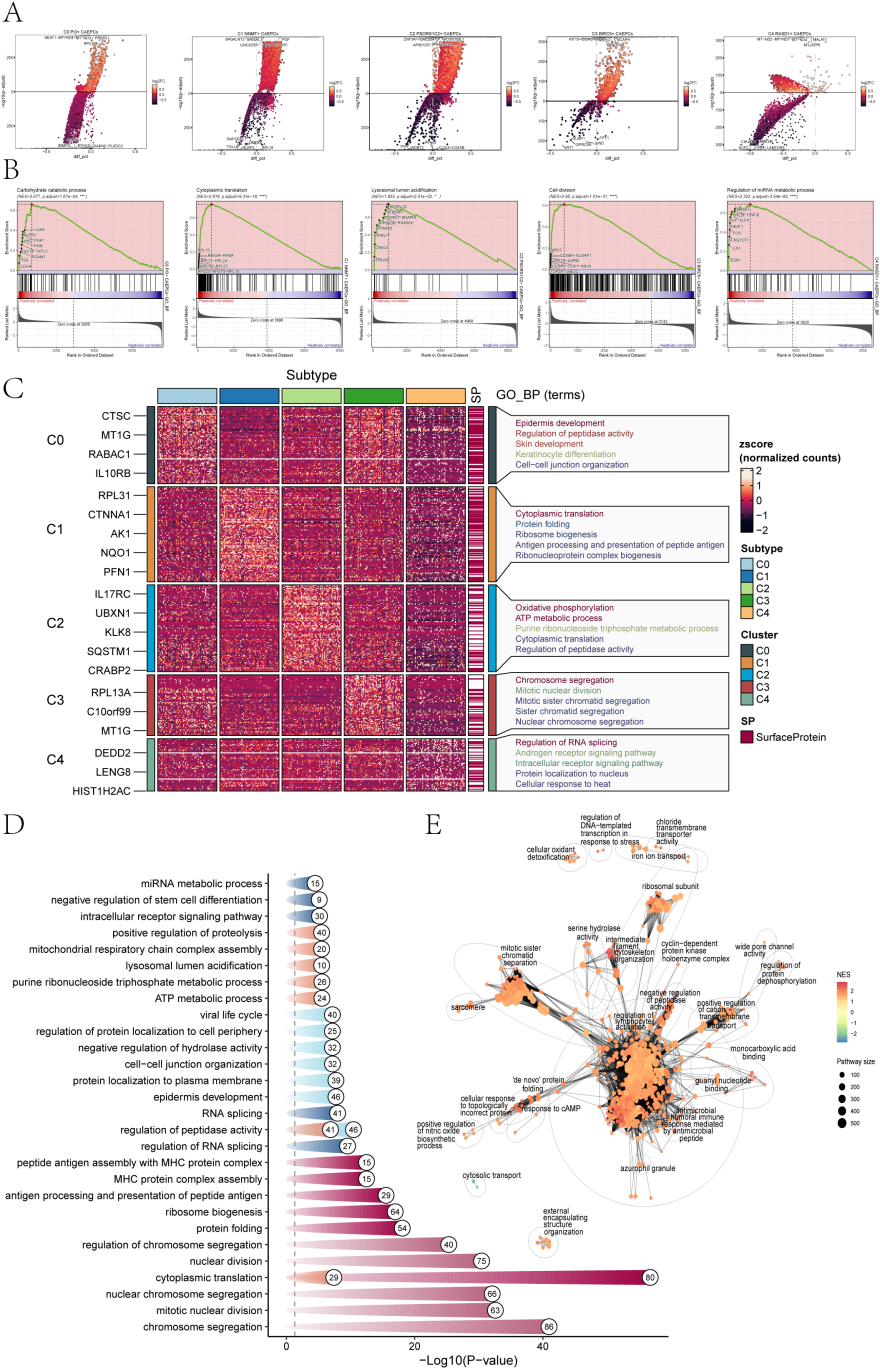
Enrichment analysis of cervical cancer subpopulations. **(A)** The top five genes that were elevated and downregulated in each of the five cervical cancer subpopulations were displayed in volcano plots. **(B)** The results of GSEA analysis highlighted only the gene sets with the highest normalized enrichment scores (NES) for each tumor subpopulation. **(C, D)** GOBP analysis was performed on the DEGs from the five cervical cancer subpopulations, presenting specific GOBP terms. **(E)** An enrichment network diagram visually represented the enrichment results of DEGs across the subpopulations (*p < 0.05, ***p < 0.001, ****p < 0.0001).

DEGs for each group were discovered in order to examine heterogeneity within the cervical cancer subpopulations. **Figure 2A** showed the top five downregulated and upregulated genes for each subpopulation. To further explore the molecular mechanisms associated with each subpopulation, we conducted several enrichment analyses using these DEGs.

Based on the GSEA results presented in **Figure 2B**, the following gene sets were found to have the highest normalized enrichment scores (NES) for the five subpopulations of cervical cancer The Carbohydrate Catabolic Process (C0, NES=2.077) highlights metabolic reprogramming, supporting rapid energy production and biosynthesis essential for tumor growth. The Cytoplasmic Translation pathway (C1, NES=2.616) reflects increased protein synthesis required for cancer cell proliferation and survival. Lysosomal Lumen Acidification (C2, NES=1.853) is associated with enhanced autophagic activity, promoting cell survival under stress. The Cell Division pathway (C3, NES=2.95) underscores the high proliferative potential of these cells, a hallmark of aggressive tumors. Lastly, the Regulation of miRNA Metabolic Process (C4, NES=2.322) indicates the critical role of miRNA dysregulation in gene expression, influencing cancer progression. These findings tie each pathway directly to mechanisms driving cervical cancer biology. The GOBP structure was depicted in **Figure 2C**, which highlighted the enrichment of DEGs in the C0 subpopulation in categories such as Skin development, Keratinocyte differentiation, Epidermis development, and Cell-cell junction organization. Cytoplasmic translation, Protein folding, Ribosome biogenesis, Antigen processing and Peptide antigen presentation, and Ribonucleoprotein complex formation were all linked to the C1 subpopulation.

For the C2 subpopulation, enrichment results highlighted processes including Oxidative phosphorylation, ATP metabolic process, Purine ribonucleoside triphosphate metabolic process, Cytoplasmic translation, and Regulation of peptidase activity. The analysis of the C3 subpopulation revealed associations with biological processes such as Chromosome segregation, Mitotic nuclear division, Mitotic sister chromatid segregation, Sister chromatid segregation, and Nuclear chromosome segregation. The C4 subpopulation showed enrichment in terms related to Regulation of RNA splicing, Androgen receptor signaling pathway, Intracellular receptor signaling pathway, Protein localization to the nucleus, and Cellular response to heat.

A thorough summary of the GOBP results and the associated p-values for each subpopulation was provided in **Figure 2D**. DEGs within CAEPCs subpopulations were found to be significantly enriched in biological processes, such as the regulation of lymphocyte activation, the negative regulation of peptidase activity, and the antimicrobial humoral immune response mediated by antimicrobial peptides, according to the enrichment analysis network diagram (**Figure 2E**).

### Trajectory analysis of CAEPCs subpopulations in cervical cancer

**Figure 3:**
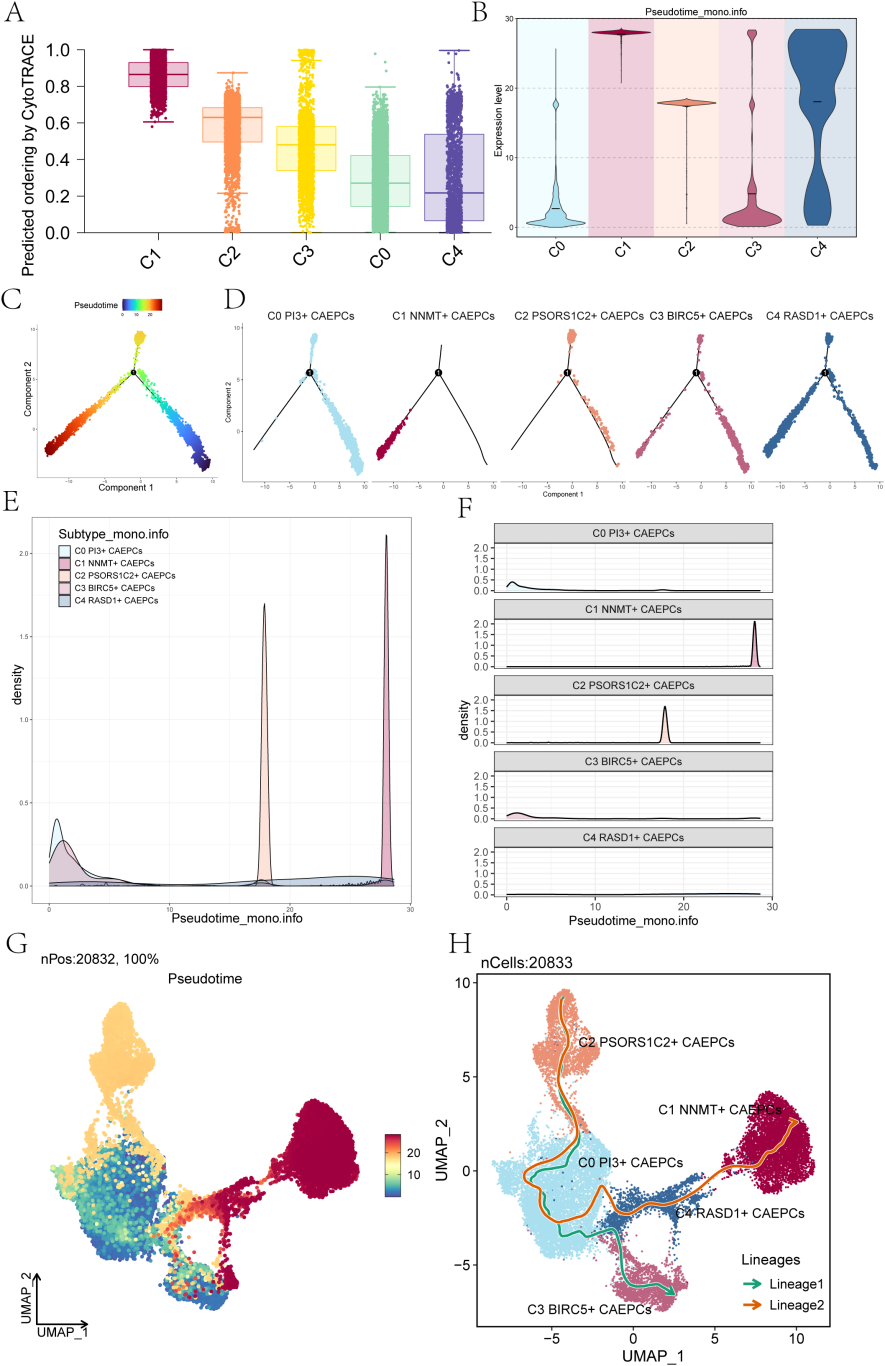
Pseudotime trajectory analysis of cervical cancer subpopulations. **(A)** The CytoTRACE scores for each cervical cancer subpopulation were presented, revealing that the C0 subpopulation had the lowest score, while the C1 subpopulation exhibited the highest. **(B)** Monocle 2 analysis illustrated the expression levels of each cervical cancer subpopulation along the pseudotime trajectory. **(C, D)** Pseudotime trajectory plots depicted the differentiation paths of cervical cancer subpopulations as predicted by Monocle, The differentiation started from the lower right corner, progressing toward the upper left and branching at state 1 into two directions: one upward and the other downward to the left. **(D)** Facet plots displayed the distribution of each cervical cancer subpopulation along the pseudotime trajectory. **(E)** Ridge plots demonstrated the density variation of cervical cancer subpopulations along pseudotime. **(F)** Ridge surface figures illustrated the specific density changes of the five cervical cancer subpopulations along the pseudotime continuum. **(G)** The UMAP plot depicted the outcomes of the pseudotime trajectory (nPos: 20,832, 100%). **(H)** Slingshot analysis identified two distinct differentiation lineages for the cervical cancer subpopulations: Lineage 1 comprised C2 PSORS1C2+ CAEPCs → C0 PI3+ CAEPCs → C3 BIRC5+ CAEPCs, while Lineage 2 consisted of C2 PSORS1C2+ CAEPCs →C0 PI3+ CAEPCs→ C4 RASD1+ CAEPCs → C1 NNMT+ CAEPCs.

In order to investigate the differentiation stemness and developmental paths of the CAEPCs subpopulations more thoroughly, we utilized CytoTRACE analysis to determine the CytoTRACE Score for every group (**Figure 3A**). According to our research, the C1 subpopulation had the highest CytoTRACE Score, whereas the C4 subpopulation had the lowest, indicating that the C1 subpopulation has a higher potential for differentiation.

According to the results of Monocle 2, C1 showed higher expression levels at the terminal stage of pseudotime than the other four subpopulations (**Figure 3B**). **Figures 3C, D** showed that the subpopulation of C1 NNMT+ CAEPCs was located in the terminal stage of the lower left branch, as indicated by the pseudotime trajectory produced by Monocle analysis. The density of the C1 NNMT+ CAEPCs subpopulation peaked in the terminal stage, as seen by the variation in density for each subpopulation along the pseudotime trajectory (**Figures 3E, F**). Additionally, we utilized Monocle analysis to generate a pseudotime trajectory UMAP plot, as shown in **Figure 3G**.

These five subpopulations followed two distinct differentiation pathways, as shown by the results of the Slingshot analysis: Lineage 1, which included C2 PSORS1C2+ CAEPCs growing into C0 PI3+ CAEPCs and eventually into C3 BIRC5+ CAEPCs; and Lineage 2, which included C2 PSORS1C2+ CAEPCs evolving into C0 PI3+ CAEPCs, then into C4 RASD1+ CAEPCs, and finally culminating in C1 NNMT+ CAEPCs (**Figure 3H**). This finding revealed that the C1 NNMT+ CAEPCs subset represented the endpoint of Lineage 2, agreeing with prior findings. Our thorough analysis focused in particular on the C1 NNMT+ CAEPCs subpopulation. According to earlier studies, benign tissues express far less NNMT than both high- and low-grade squamous intraepithelial lesions (57). Based on the analysis of single-cell sequencing data and previous studies, we further investigated the key subpopulation, C1 NNMT+ CAEPCs.

### Transcription factors and cell interactions in cervical cancer subgroups

**Figure 4:**
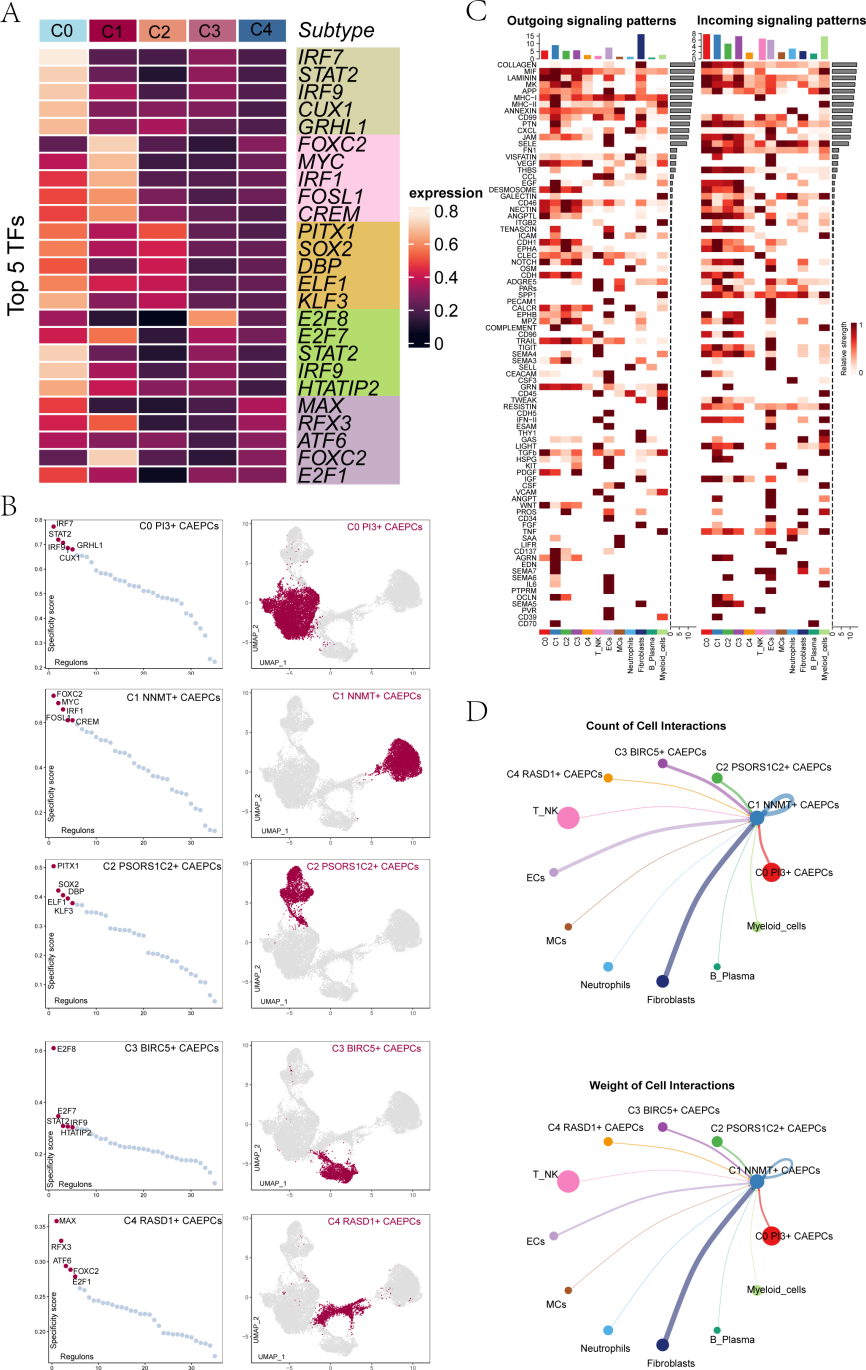
Gene regulatory networks and cell interactions among cervical cancer subgroups. **(A)** Heatmap displayed the top five transcription factors in each cervical cancer tumor subgroup. **(B)** Transcription factors within each subgroup were ranked based on RSS, with UMAP plots illustrating their distribution. **(C)** A heatmap showing the incoming (right) and outgoing (left) signaling patterns of different cervical cancer cell types. **(D)** Focusing on C1 NNMT+ CAEPCs, this panel presented cell interaction counts (top) and interaction weights (bottom).

To determine subgroup-specific transcription factors, we used pySCENIC to delineate gene regulatory networks. The five TFs with the highest activity for each subgroup were C0 PI3+ CAEPCs (IRF7), C1 NNMT+ CAEPCs (FOXC2), C2 PSORS1C2+ CAEPCs (PITX1), C3 BIRC5+ CAEPCs (E2F8), and C4 RASD1+ CAEPCs (MAX) (**Figure 4A**). **Figure 4B** highlighted subgroup distributions in UMAP plots and displayed RSS-based TF rankings. The signaling strengths for each subgroup were shown in **Figure 4C**. Cell interaction weights and counts showed a strong correlation with fibroblasts in **Figure 4D**, which was centered on the C1 NNMT+ CAEPCs subgroup.

### Construction and validation of the NCRS model

**Figure 5:**
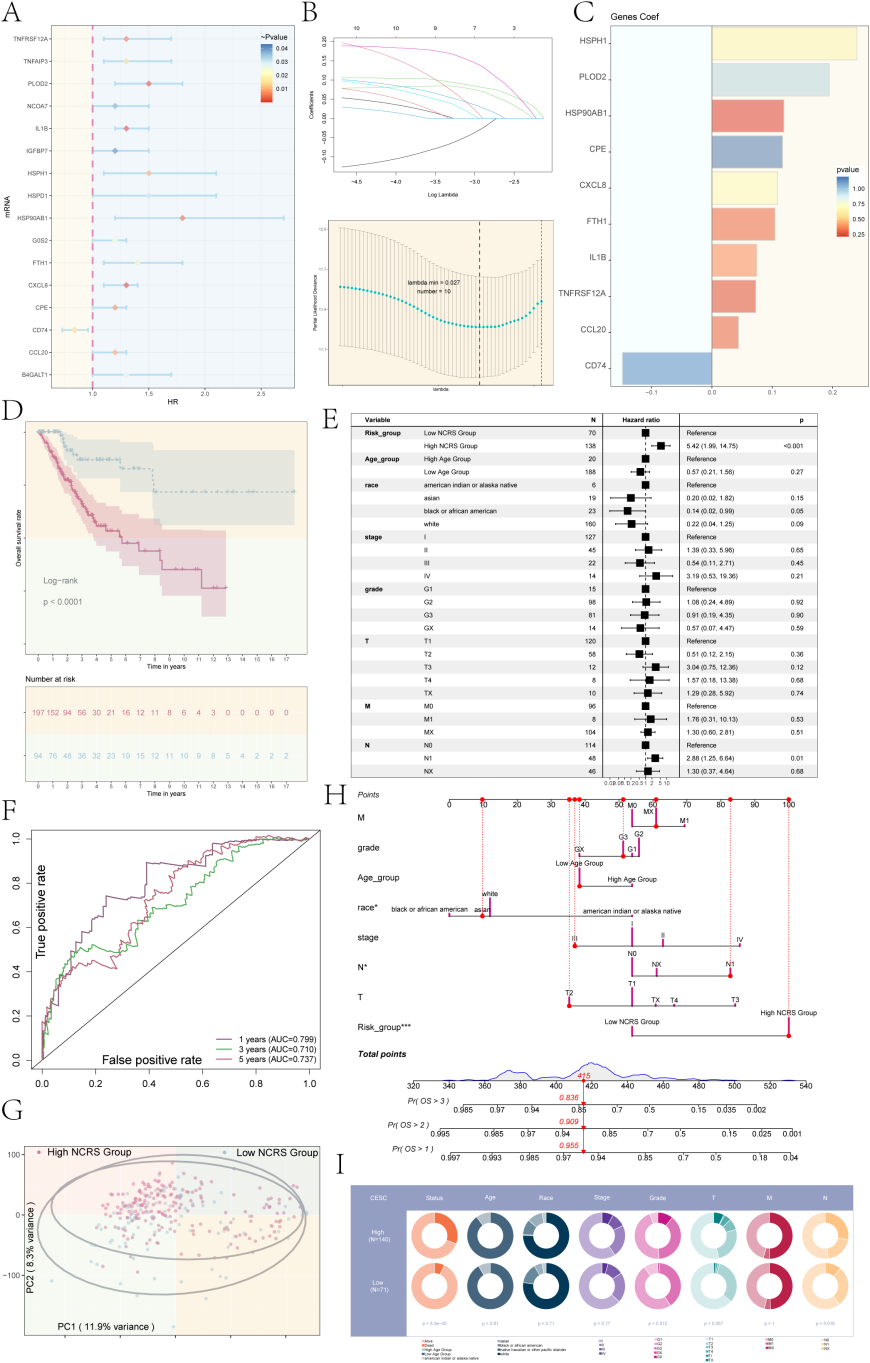
Development and validation of a novel cervical cancer model. **(A)** Univariate Cox regression analysis identified the top 100 marker genes associated with the key subpopulation C1 NNMT+ CAEPCs. **(B)** An optimal prognostic model was developed using LASSO regression, resulting in the selection of 10 genes at a lambda.min of 0.027. **(C)** The coefficient values for each prognostic gene were illustrated in a bar graph. **(D)** Kaplan-Meier survival curves were employed to compare survival outcomes across the distinct NCRS groups. **(E)** Results from the multivariate Cox regression analysis, which incorporated NCRS along with other clinical variables (age, race, stages, grades, and TNM staging of cervical cancer patients), were presented in a forest plot. **(F)** ROC curve analysis for the NNMT CAEPCs Risk Score (NCRS) was presented, highlighting the AUC values for 1-, 3-, and 5-year survival predictions. **(G)** A scatter plot generated from principal component analysis distinguished between various NCRS groups. **(H)** A nomogram was created to predict overall survival (OS) at 1, 2, and 3 years based on age, race, stages, TNM staging, and NCRS group classifications. **(I)** Pie chart illustrated the distribution of clinical data (e.g., status, age, race, tumor stage) across high and low NCRS groups in the CESC cohort. (*P < 0.05, ***P < 0.001).

In order to determine the predictive importance of the top 100 marker genes in the important subpopulation (C1 NNMT+ CAEPCs), we conducted a univariate Cox regression analysis (**Figure 5A**). 16 genes were found to be associated with patient survival outcomes by this investigation. To address multicollinearity, we performed Least Absolute Shrinkage and Selection Operator (LASSO) regression on the genes (**Figure 5B**). The model identified 10 prognostic-related genes (CD74, HSPH1, CXCL8, CPE, HSP90AB1, PLOD2, TNFRSF12A, FTH1, IL1B, and CCL20) and obtained optimal performance at lambda.min = 0.027. We then used multivariate Cox regression analysis to compute the coefficients for each gene. We used the obtained prognostic-related gene coefficient values (**Figure 5C**) to produce the NNMT CAEPCs Risk Score (NCRS) using the following algorithm: NCRS (C1 NNMT+ CAEPCs Risk Score) = CD74 * (-0.148426201124091) + HSPH1 * 0.240402828176152 + CXCL8 * 0.108590186774839 + CPE * 0.117113706206308 + HSP90AB1 * 0.119471572156487 + PLOD2 * 0.194498510649581 + TNFRSF12A * 0.0727111252257242 + FTH1 * 0.104526867983822 + IL1B * 0.0744859997278779 + CCL20 * 0.0440648251742935). Based on the NCRS, patients were categorized into groups with High and Low NCRS; **Figure 5D** displayed the survival statistics for different groups. As predicted, the High NCRS group had a much worse outcome (P < 0.0001).

For the 1-year, 3-year, and 5-year forecasts, respectively, the ROC curve analysis for the NCRS revealed AUC values of 0.799, 0.710, and 0.737 (**Figure 5F**). As shown in **Figure 5E**, we conducted univariate Cox regression analysis for patients with cervical cancer, taking into account the NCRS as well as clinical factors such age, stages, grades, and TNM staging. The forest plot displayed a hazard ratio (HR) of 5.42 for different NCRS categories, with a 95% confidence interval (CI) of 1.99 to 14.75 (P < 0.001).Moreover, principal component analysis for the different NCRS groups showed that the variance for PC1 was 11.9% and for PC2 was 8.3%, as shown in **Figure 5G**. To predict the 1-, 2-, and 3-year Overall Survival (OS) of patients with cervical cancer, we developed a nomogram incorporating age, race, stages, TNM staging, and NCRS classifications (**Figure 5H**). Race and NCRS categories were important factors in determining OS scores. **Figure 5I** showed the clinical features (e.g., status, age, race, and tumor stage) that we compared between the high and low NCRS groups in the CESC cohort. According to the results, the high NCRS group had a noticeably greater percentage of dead status cases than the low NCRS group.

### Prognostic-related gene analysis in NCRS

**Figure 6:**
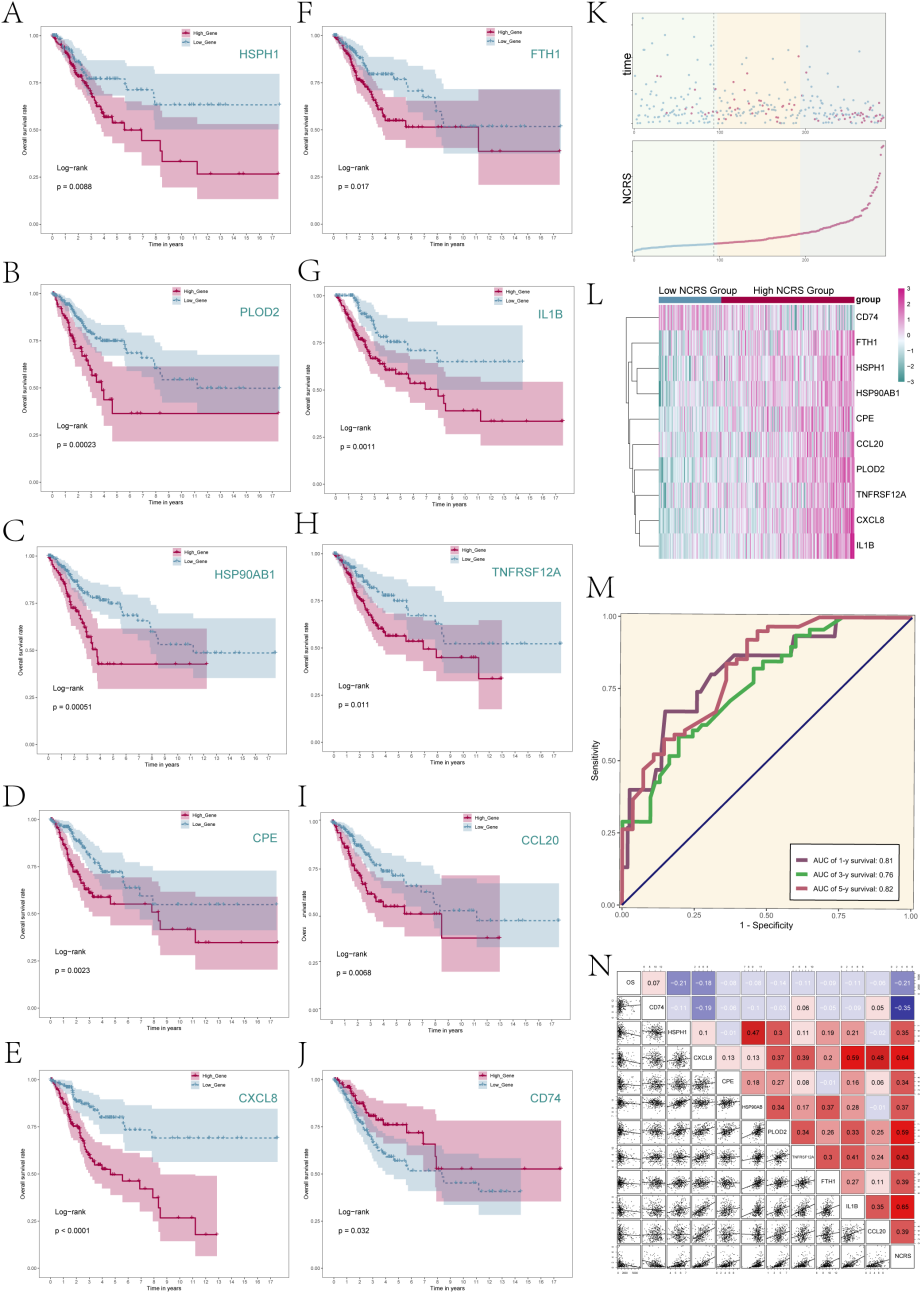
NCRS model related analysis. **(A–J)** Survival analysis was performed to compare the prognosis between the high and low expression groups of the ten prognostic genes comprising the NCRS. **(K)** The scatter plots (top) depicted the survival status over time for the various NCRS groups, while a curve illustrated the NNMT CAEPCs Risk Score for both groups (bottom). **(L)** The differential expression of NCRS-related prognostic genes across the various NCRS groups was illustrated. **(M)** There were reported AUC values of 0.81, 0.76, and 0.82 for the 1-, 3-, and 5-year predictions, respectively. **(N)** Correlation analysis results among NCRS prognostic genes, overall survival (OS), and NCRS were displayed through scatter plots and a heatmap.

The predictive characteristics of the ten genes linked to prognosis were then assessed. Patients were divided into High and Low Gene Groups according to the median levels of gene expression. Following survival studies for these expression groups, corresponding Kaplan-Meier survival curves (**Figures 6A–J**) were produced; all of these showed statistical significance (P < 0.05). The results showed that whereas the remaining nine genes had better prognoses in the low expression group, the CD74 high expression group had a better prognosis than the CD74 low expression group. This was consistent with earlier research results showing that CD74 was a protective gene while the other nine genes were risk genes (HSPH1, CXCL8, CPE, HSP90AB1, PLOD2, TNFRSF12A, FTH1, IL1B, and CCL20).

The survival outcomes of the Low NCRS Group and High NCRS Group were then comprehensively analyzed (**Figure 6K**). **Figure 6L** presented the difference expression levels of the 10 prognostic-related genes that make up the NCRS between the groups, whereas **Figure 6K** displayed the survival status throughout time and the NNMT CAEPCs Risk Score. The results suggested that CD74 may have had higher expression levels in the Low NCRS Group, while the other nine prognostic-related genes were likely more expressed in the High NCRS Group.

**Supplementary Figures S3A–J** demonstrated the variations in the expression levels of the 10 prognostic-related genes between the various NCRS groups. The ROC curve for the NCRS model was displayed in **Figure 6M**. The AUC values for 1, 3, and 5 years are 0.81, 0.76, and 0.82, respectively, suggesting good prediction accuracy at different time points. Furthermore, as indicated in **Figure 6N**, we looked at the relationships between the prognostic-related genes in the NCRS, Overall Survival (OS), and NCRS scores. Analysis revealed that the prognostic gene CD74 was negatively correlated with most other prognostic genes and NCRS. In contrast, the other nine prognostic genes—HSPH1, CXCL8, CPE, HSP90AB, PLOD2, TNFRSF12A, FTH1, IL1B, and CCL20—were positively correlated with NCRS.

### Immune infiltration analysis of different NCRS groups

**Figure 7:**
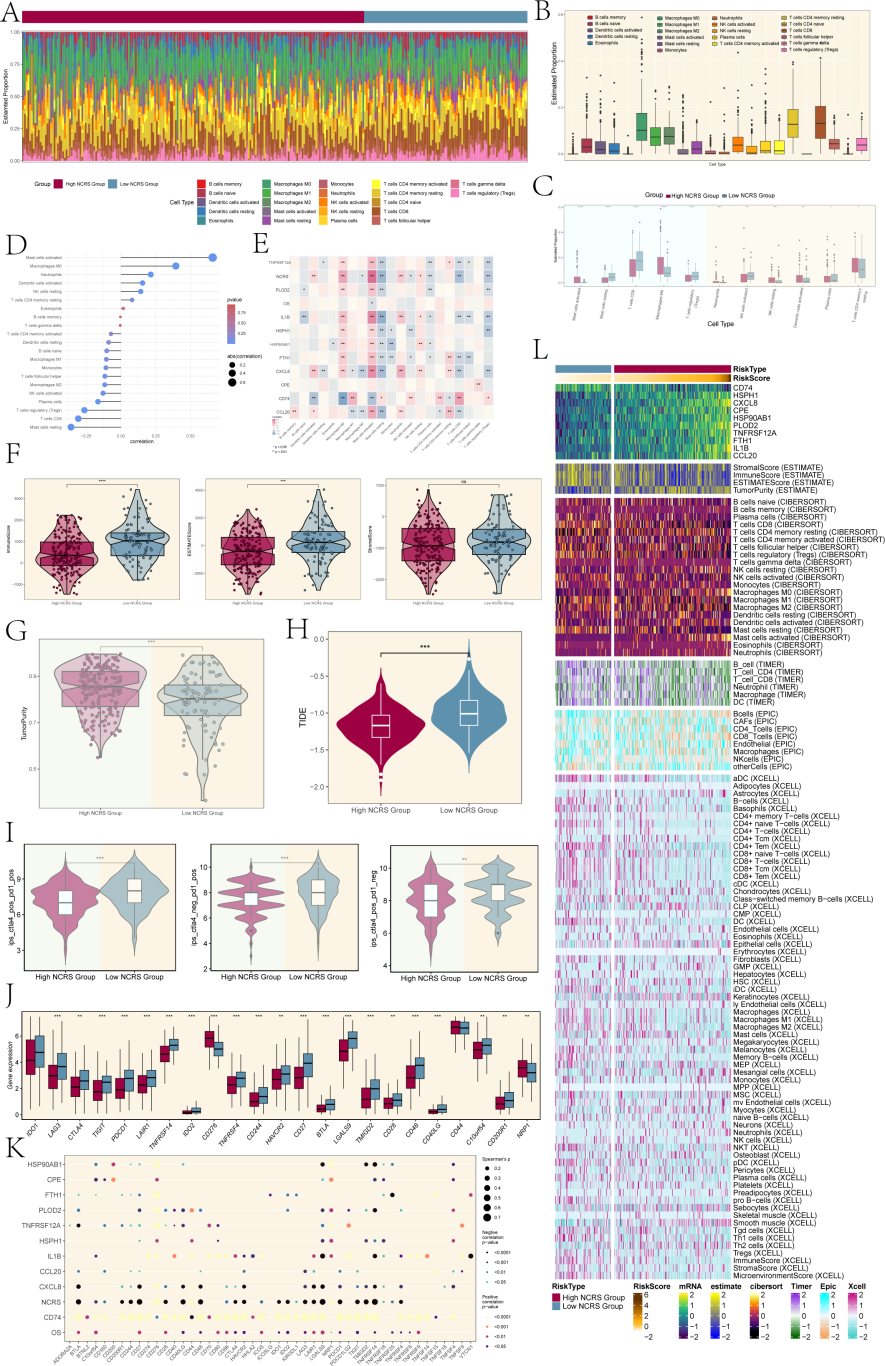
Immune infiltration analysis. **(A)** The proportions of 22 infiltrating immune cell types across various NCRS groups were visualized using a stacked bar graph. **(B)** The estimated proportions of these immune infiltrates across the total sample were represented in boxplots. **(C)** Boxplots highlighted immune infiltration cells with statistically significant differences across the various NCRS groups. **(D)** The association between different kinds of immune infiltration cells and NCRS was illustrated using a lollipop plot. **(E)** A heatmap was generated to illustrate the correlation between immune infiltration cells, overall survival (OS), and NCRS-related prognostic genes. **(F)** Significant differences were found in the Immune and ESTIMATE Scores when comparing the ESTIMATE, Immune, and Stromal scores between the various NCRS groups. **(G)** Tumor purity levels were assessed and compared between the various NCRS groups. **(H)** TIDE values for both NCRS groups were presented in boxplots. **(I)** Treatment responses to CTLA-4 and PD-1 were illustrated in boxplots, highlighting differences between the various NCRS groups. **(J)** Boxplots were employed to compare the expression levels of immune checkpoint genes across the various NCRS groups. **(K)** A spot plot depicted the correlation among NCRS-related prognostic genes, OS, NCRS, and immune checkpoint genes. **(L)** A heatmap, calculated using various algorithms, displayed the expression of immune infiltration cells and immune scores across the various NCRS groups. (*P < 0.05, **P < 0.01, ***P < 0.001, ****P < 0.0001; ‘ns’ indicated non-significance.).

Tumor invasion, metastasis, and tumor development are significantly influenced by cells in the tumor microenvironment. Based on our investigation of the Tumor Immune Microenvironment (TIME) in the different NCRS groups, **Figure 7A** illustrated the distribution of twenty-two immune cell types in both High and Low NCRS Groups. **Figure 7B** indicated the makeup of immunological cells in the sample, with resting CD4 memory T cells, CD8 T cells, and macrophages making up the majority of the cell population. The immune cell expression levels that were different across the groups were presented in **Figure 7C**. While T cells CD4 memory resting and Macrophages M0 were more common in the High NCRS Group, Mast cells resting, T cells CD8, and T cells regulatory were higher in the Low NCRS Group.

A positive correlation between NCRS scores and Mast cells activated and Macrophages M0 was demonstrated by the lollipop plot presented in **Figure 7D**. The relationships between immune cells, prognostic-related genes, overall survival (OS), and NCRS were shown in **Figure 7E** along with P values. The findings demonstrated that CD74 was negatively correlated with Macrophages M0 and activated Mast cells, while most prognostic-related genes showed positive associations. Except for a positive correlation with CD74, resting Mast cells were negatively correlated with the majority of prognostic-related genes.

In order to determine if stromal and immune cells were present in the tumor microenvironment, we additionally assessed the Stromal Score, Immune Score, and ESTIMATE Score for different NCRS groups using the ESTIMATE algorithm (**Figure 7F**). The Stromal scores between the two groups did not show significant differences; however, the Low NCRS Group exhibited elevated Immune and ESTIMATE scores, indicating a higher level of immune cell infiltration and a more active tumor microenvironment. This may impact their response to immunotherapies and potentially influence patient prognosis. The High NCRS Group had considerably greater levels, according to the tumor purity assessment (**Figure 7G**). This analysis indicated that tumor samples from the High NCRS Group exhibited a greater proportion of tumor cells and a reduced presence of non-tumor components, such as immune cells and stromal elements. In order to evaluate tumor immunological dysfunction and exclusion, which suggests possible immune evasion, we examined immune cell exclusion and immune dysfunction within the tumor microenvironment. The TIDE values presented in **Figure 7H** indicated that the High NCRS Group had lower scores compared to the Low NCRS Group. This observation suggests a potential increased responsiveness of the High NCRS Group to immunotherapies, including immune checkpoint inhibitors, warranting further investigation.

The High NCRS Group exhibited lower scores for the treatment modalities of CTLA4-positive PD1-positive, CTLA4-negative PD1-positive, and CTLA4-positive PD1-negative, in contrast to the Low NCRS Group (**Figure 7I**). A robust correlation exists between the efficacy of immunotherapy and the NCRS score, evidenced by the heightened responsiveness of the High NCRS Group to CTLA4 and PD1-targeted treatments. In a similar vein, the Low NCRS Group exhibited elevated expression levels of most genes linked to immune checkpoints (**Figure 7J**). In contrast, the High NCRS Group demonstrated increased expression of genes such as CD276 and NRP1.

This implied that the various NCRS groups had distinct immune-suppressive mechanisms, highlighting the importance of individualized treatment plans to account for these differences.

The relationships between immunological checkpoint-related genes and the prognostic-related genes that make up the NCRS, OS, and NCRS were shown in **Figure 7K**. The majority of immune checkpoint-related genes and CD74 had a positive connection, but many of these genes showed a negative correlation with the NNMT CAEPCs Risk Score (NCRS). The NCRS’s particular function in the immunological escape mechanism was further supported by its negative correlation. Lastly, we assessed immune cell infiltration and immunological scores between the High and Low NCRS Groups using the ESTIMATE, CIBERSORT, XCELL, and EPIC algorithms (**Figure 7L**).

### Immune enrichment analysis and mutation sensitivity analysis

**Figure 8:**
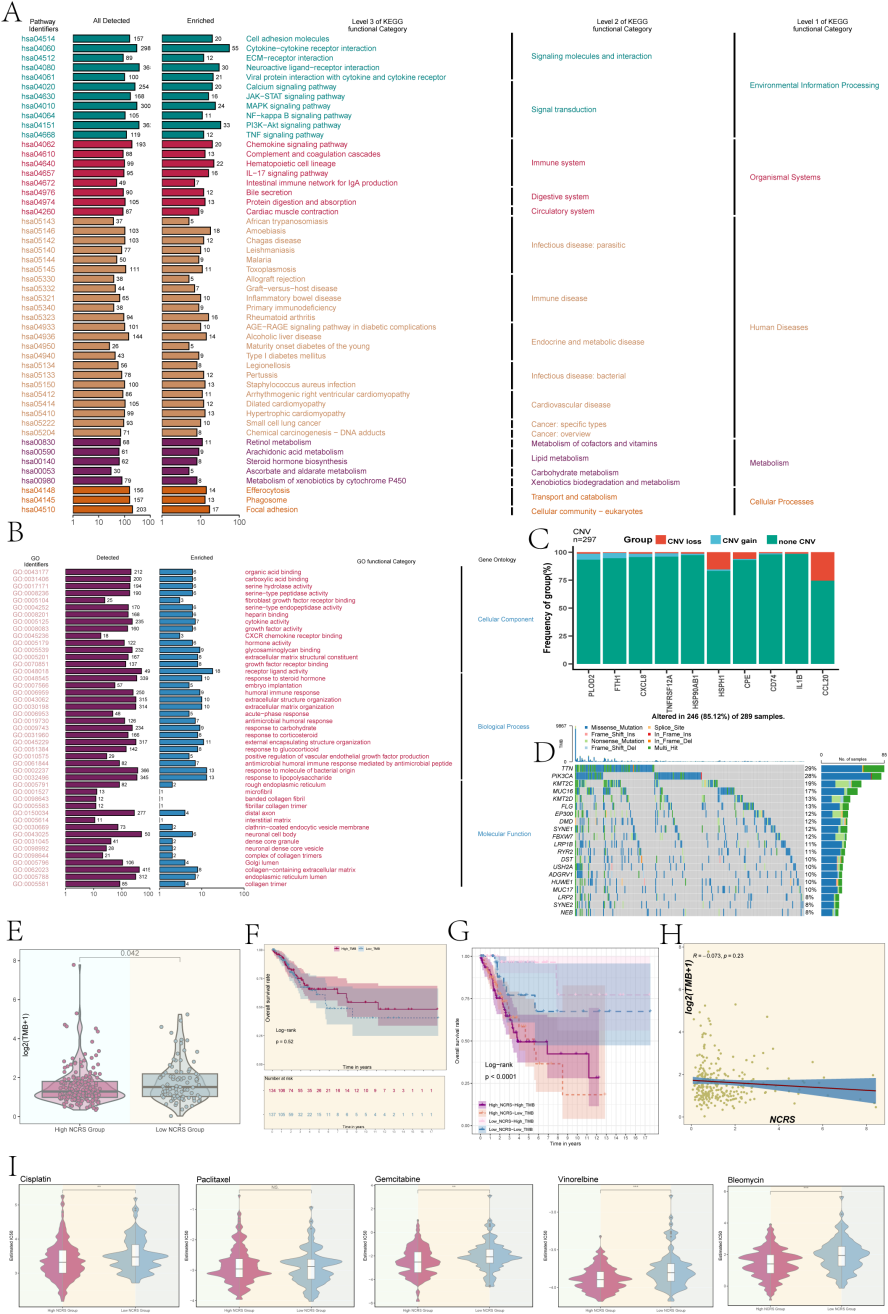
Enrichment analysis, TMB, and drug sensitivity analysis. **(A, B)** Based on DEGs, we conducted KEGG enrichment and Gene Ontology analysis of different NCRS groups. **(C)** A bar graph was used to show the copy number variation (CNV) of the prognostic genes that make up the NCRS. Blue showed CNV gains, red indicated CNV losses, and green indicated no change. **(D)** A gene mutation waterfall plot illustrated the top 20 most mutation frequencies genes. **(E)** Boxplot revealed a significant difference in tumor mutational burden (TMB) values among the different NCRS groups (P = 0.042). **(F)** A P value of 0.52 was obtained from the survival analysis for the High and Low TMB Groups. **(G)** A significant P value of less than 0.0001 was found in the survival analysis results for the High NCRS-High TMB, High NCRS-Low TMB, Low NCRS-High TMB, and Low NCRS-Low TMB Groups. **(H)** An evaluation of the correlation between the NCRS and TMB values produced a R value of -0.073 and a P value of 0.23. **(I)** Drug sensitivity analysis results were conducted in the various NCRS groups for cisplatin, gemcitabine, vinorelbine, bleomycin, and paclitaxel. (**P < 0.01, ***P < 0.001, ‘ns’ indicated non-significance).

To evaluate the immunological enrichment status across the different NCRS groups, we conducted KEGG enrichment analysis. The findings revealed significant enrichment in biological processes related to cytokine-cytokine receptor interactions and neuroactive ligand-receptor interactions (**Figure 8A**). Significant activity in biological processes, such as receptor-ligand activity, response to bacterial compounds, and response to lipopolysaccharide, was found by GO enrichment analysis (**Figure 8B**). The ten prognostic-related genes’ Copy Number Variation (CNV) events were analyzed, and the results showed that while most genes did not exhibit CNV events, HSPH1, CPE, and CCL20 experienced CNV loss events (**Figure 8C**). Further research into the biological mechanisms and clinical significance of these genes is therefore required, as the reduction in copy number may have led to diminished or lost expression of these genes, thereby leading to carcinogenesis and progression. We determined which genes were mutated most frequently and tracked the development of the top 20. Remarkably, TTN had the highest mutation rate (29%), with PIK3CA following closely after (28%) (**Figure 8D**).

Additionally, we evaluated the Tumor Mutational Burden (TMB) between the groups and found that the Low NCRS Group had a higher TMB value (P=0.042), indicating that the mutation features in this group may be richer, which could affect how this group responds to immunotherapy (**Figure 8E**). Survival prognosis showed no statistically significant difference between the High and Low NCRS groups (**Figure 8F**). By combining the NCRS and TMB, the patients were divided into four groups: Low NCRS-High TMB group, Low NCRS-Low TMB group, High NCRS-High TMB group, and Low NCRS-Low TMB group. The High NCRS-Low TMB group had the worst prognosis, according to survival analysis (**Figure 8G**). This group’s lower tumor mutational burden and higher NCRS score may have made it easier for the tumor to evade immune surveillance because fewer tumor neoantigens were detected by the immune system. In contrast, the Low NCRS-High TMB group demonstrated a better prognosis, which may be related to the less immunosuppressive tumor microenvironment. This favorable combination may enhance immune surveillance and anti-tumor immune responses, contributing to improved outcomes and greater sensitivity to immunotherapy. No significant linear correlation was identified between TMB values and NCRS scores (**Figure 8H**).

Ultimately, a comparison of the reactions to chemotherapeutic drugs among the various NCRS groups showed notable variations (**Figure 8I**). The calculated IC50 value for cisplatin, bleomycin, vinorelbine, and gemcitabine was found to be lower in the High NCRS Group, indicating that these individuals may be more susceptible to the effects of these medications. These results suggest that the NCRS score may serve as a biomarker for predicting chemotherapy efficacy, thereby aiding in the formulation of personalized treatment strategies.

### GSVA analysis

**Figure 9:**
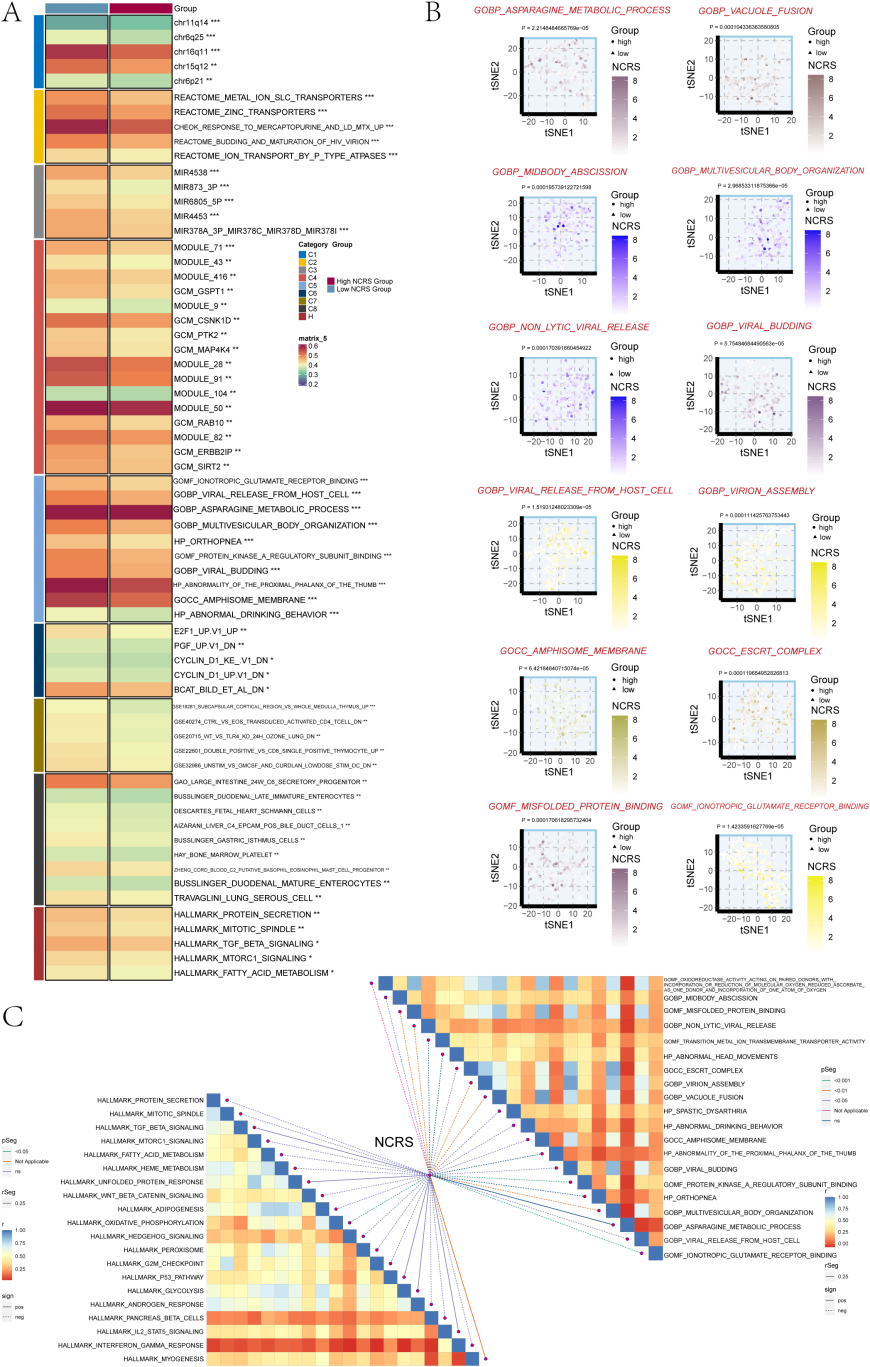
GSVA analysis. **(A)** The two NCRS Groups underwent GSVA analysis using the Molecular Signatures Database (MsigDB). **(B)** GO pathway activity differences between the various NCRS groups were shown using t-SNE plots. **(C)** To evaluate the association between NCRS and the GOBP, GOMF, GOCC, HP, and HALLMARK gene sets, a Spearman correlation analysis was performed. (*P < 0.05, **P < 0.01, ***P < 0.001, ‘ns’ indicated non-significance).

We used the Molecular Signatures Database (MsigDB) to perform Gene Set Variation Analysis (GSVA) in order to investigate the biological features associated with the NCRS score in the TCGA dataset. The biological characteristics of different groups were depicted in this analysis (**Figure 9A**). The asparagine metabolic process, non-lytic viral release, viral budding, viral release from host cells, virion assembly, amphisome membrane, and misfolded protein binding were among the biological processes in which the High NCRS Group showed considerable enrichment (**Figure 9B**). We also looked at the connection between biological functions and NCRS scores. An algorithm that found gene set overlaps in other MSigDB datasets and kept genes with coordinated expression was used to create the Hallmark gene sets. The NCRS score revealed statistically significant positive correlations with biological processes such as GOMF oxidoreductase activity acting on paired donors with incorporation or reduction of molecular oxygen reduced ascorbate, GOCC virion assembly, GOMF misfolded protein binding and GOMF transition metal ion transmembrane transporter activity as well as Hallmark gene sets like HEME METABOLISM, ADIPOGENESIS and GLYCOLYSIS (**Figure 9C**). These findings underscore HPV infection as a significant causative factor in cervical cancer, revealing a strong correlation between the high NCRS group and viral activity.

### *In vitro* experimental validation

**Figure 10:**
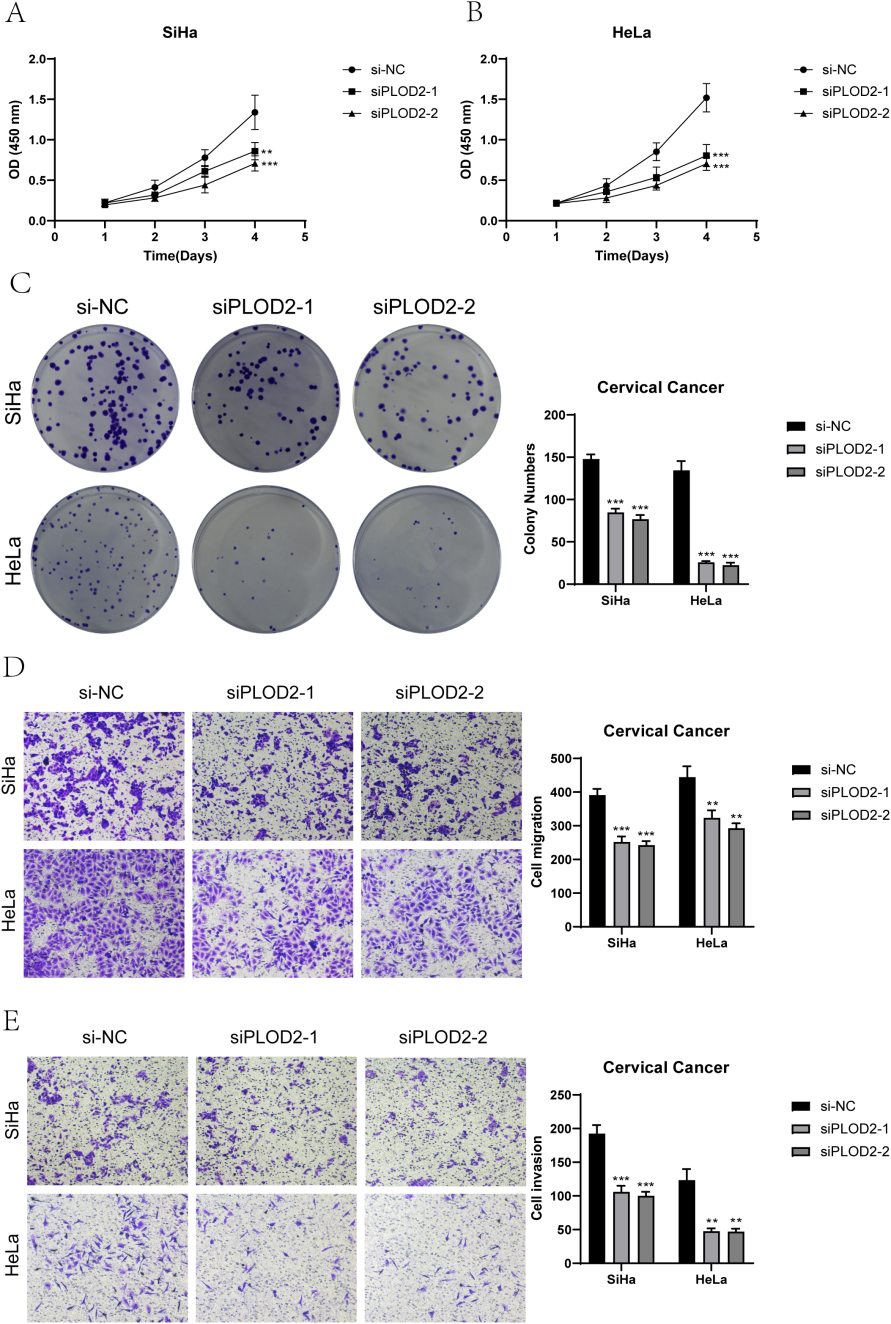
PLOD2 Knockdown effects. **(A, B)** The CCK-8 assay demonstrated that the knockdown of PLOD2 significantly compromised the proliferative capacity of both SiHa and HeLa cell lines. **(C)** The plate colony formation assay demonstrated a marked reduction in the colony-forming ability of cervical cancer cell lines subsequent to PLOD2 knockdown. **(D, E)** Transwell assays showed that PLOD2 significantly reduced the migration and invasion abilities of the two cell lines. (** P<0.01, *** P<0.001).

**Figure 11:**
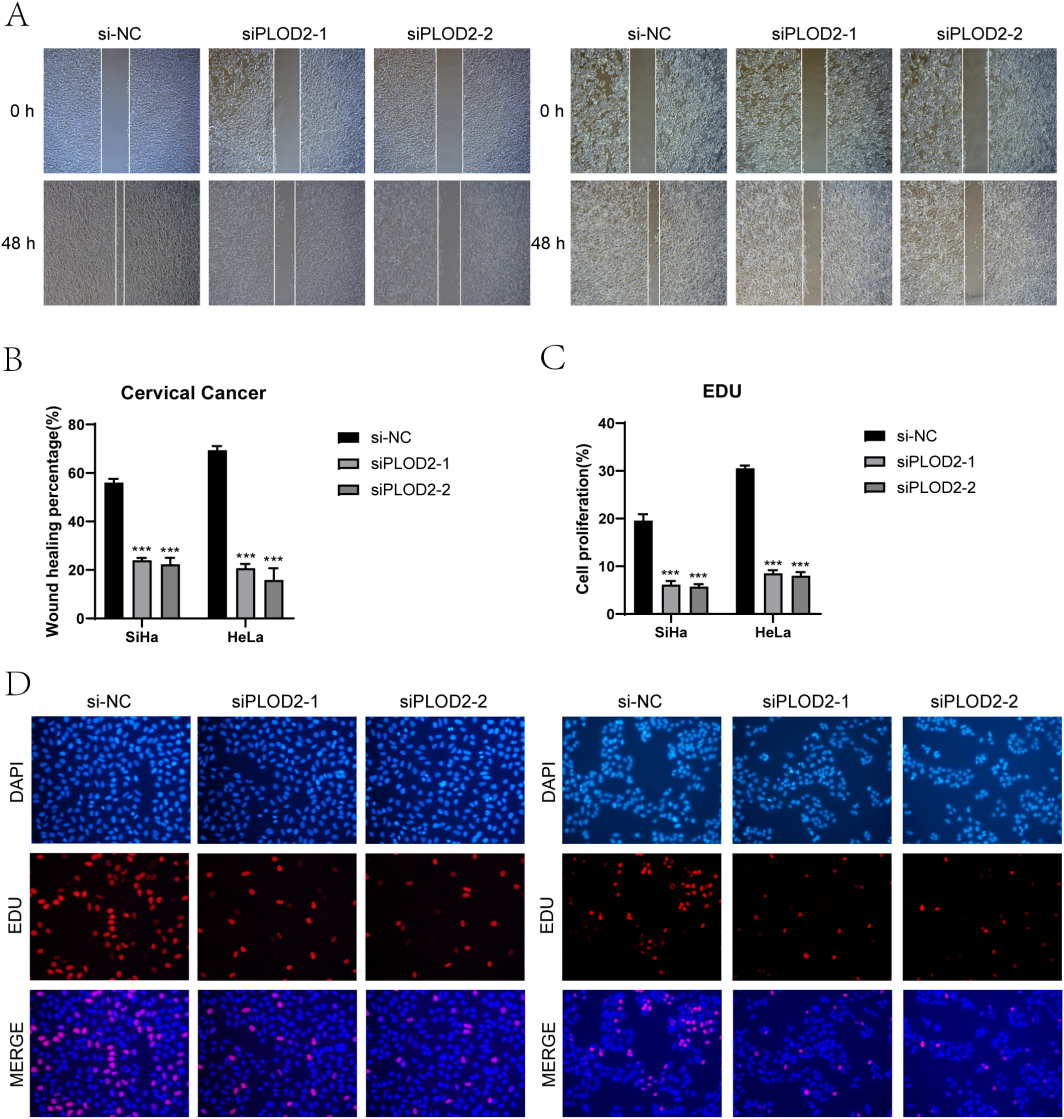
Effects on migration and proliferation. **(A, B)** Results from the scratch assay demonstrated that migration of SiHa and HeLa cell lines was significantly hindered following PLOD2 knockdown. **(C, D)** Following PLOD2 knockdown, EdU staining revealed a significant reduction in the proliferative ability of HeLa and SiHa cell lines. (***P < 0.001).

We conducted *in vitro* research to shed further light on the prognostic gene PLOD2’s function in cervical cancer. Two cervical cancer cell lines, SiHa and HeLa, along with a negative control group, were selected (**Figures 10**, **11**). When comparing the two cell lines with PLOD2 knockdown to the control group, CCK-8 assays showed a considerably lower viability (**Figures 10A, B**). PLOD2 gene silencing dramatically decreased the quantity and size of colonies produced by cervical cancer cell lines, as shown by plate cloning tests (**Figure 10C**). **Figures 10D, E** from the Transwell assays confirmed that the knockdown of PLOD2 significantly hindered the migration and invasion of cervical cancer cells. The wound healing experiment showed that, in comparison to the negative control group, PLOD2 knockdown in SiHa and HeLa cell lines resulted in significantly bigger wound widths after 48 hours (**Figures 11A, B**). Cell proliferation assays demonstrated a marked decrease in the number of cells in PLOD2 knockdown SiHa and HeLa lines when compared to the negative control group (P < 0.001) (**Figures 11C, D**). A thorough analysis of the experimental data revealed a marked reduction in both the migratory and proliferative capacities of cells with PLOD2 knockdown. This finding indicates that PLOD2 is integral to cervical cancer cell behavior, likely facilitating tumor progression by promoting cell migration and proliferation. These findings highlight PLOD2 as a potential therapeutic target in cervical cancer, warranting further investigation into its underlying mechanisms to better understand the disease’s progression.

## Discussion

To investigate the heterogeneity and tissue distribution of cervical cancer cells, we performed an in-depth analysis of single-cell sequencing data obtained from the Array Express database. Using marker genes, we classified high-quality cells into eight distinct types: EPCs, ECs, MCs, B Plasma, T NK, Myeloid cells, Fibroblasts, and Neutrophils. The UMAP visualization effectively illustrated the distribution of these cell types and their tissue origins. Notably, EPCs were predominantly present in T1 and T3 samples, while ECs from HSIL and tumor tissues primarily originated from the T2 sample. This tissue-specific distribution offers insights into the potential roles of these cell types in cervical cancer progression. Further investigation of the cell cycle revealed that a subset of EPCs was in the G2M phase, indicative of a strong proliferative capacity. By integrating G2M Score and Cell Stemness AUC analysis, we identified a subgroup of EPCs with high stemness traits. This finding underscores the critical role of EPCs in the tumor microenvironment, emphasizing their potential contribution to tumor progression and drug resistance.

The pivotal role of the C1 subpopulation in the differentiation process was underscored by CytoTRACE and Monocle analyses, emphasizing its crucial involvement in tumor evolution. This subpopulation was situated at the end of Slingshot Lineage 2 and the terminal stage of the Monocle 2 trajectory, exhibiting the highest CytoTRACE Score. Its significant differentiation capacity and unique differentiation mechanisms suggest that it could be a valuable target for therapeutic intervention. Previous studies have identified NNMT as a key metabolic regulator in the differentiation of cancer-associated fibroblasts (CAFs) in the tumor stroma, emphasizing its therapeutic potential and its crucial role in cancer progression (58). This conclusion emerged from proteomic analyses of patients with HGSC. Additionally, previous research has revealed that the NNMT-DNMT1 axis plays a critical role in preserving the susceptibility of cancer cells to inhibition of oxidative phosphorylation (59). In high-grade squamous intraepithelial lesions and normal cervical cells, PLOD2 expression is higher in squamous cell carcinoma (60). Our investigation into C1 NNMT+ CAEPCs was motivated by the relatively unexplored role of NNMT in cervical cancer.

We developed a model known as the NNMT CAEPCs Risk Score (NCRS) and conducted a comprehensive evaluation of its predictive capabilities for patients with cervical cancer. By employing univariate Cox regression alongside LASSO regression analyses, we identified ten genes—CD74, HSPH1, CXCL8, CPE, HSP90AB1, PLOD2, TNFRSF12A, FTH1, IL1B, and CCL20—that exhibited strong correlations with prognosis. Among these, nine genes were classified as risk factors, including TNFRSF12A, FTH1, CXCL8, CPE, HSP90AB1, PLOD2, and CCL20, while CD74 was identified as a protective gene. Notably, HSPH1 exhibited the highest coefficient value of 0.24 among the risk genes, closely followed by PLOD2 at 0.19. The results demonstrated that patients categorized in the high NCRS group had markedly poorer prognoses than those in the low NCRS group, thereby affirming NCRS as a significant prognostic factor in cervical cancer.

The ROC curve analysis for the NCRS model demonstrated robust predictive accuracy for outcomes at one, three, and five years. Furthermore, NCRS was confirmed as an independent prognostic predictor in multivariate Cox regression analysis that included clinical variables. This underscores the significance of NCRS in the prognostic classification of cervical cancer.

The TME significantly impacts tumor growth and treatment efficacy (61). Most solid tumors exhibit immunosuppressive characteristics, as evidenced by extensive research on the TME (62). Our examination of immune infiltration revealed significant variations in the distribution of immune cell types across the NCRS groups. Specifically, the low NCRS group displayed a greater density of stromal and immune cells, while the high NCRS group demonstrated enhanced tumor cell purity. This observation suggests that the high NCRS group may employ more aggressive immune evasion strategies.

Clinical trials have demonstrated that Zalifrelimab (anti-CTLA-4) and Balstilimab (anti-PD-1) possess acceptable safety profiles and induce sustained responses, highlighting their potential as effective therapies for advanced cervical cancer (63). Additionally, our analysis of immune checkpoint-related genes revealed that the high NCRS group demonstrated a more favorable response to immune checkpoint inhibitors, particularly when treated with combinations of CTLA4 and PD1 inhibitors. This finding suggests that patients in this group may experience enhanced benefits from immunotherapy.

Immune enrichment analyses (KEGG and GO) revealed significant enrichment of pathways related to immune evasion and viral activity, including “GOBP viral release from host cell” and “GOCC virion assembly,” in the high NCRS group. These findings reinforce the unique role of NCRS within the cervical cancer immune landscape, closely associated with HPV infection, the primary pathogenic factor in cervical cancer.

*In vitro* experiments validated the impact of PLOD2, a key gene within the NCRS, on the biological activity of cervical cancer cells. Prior studies have shown that PLOD2 is expressed at higher levels in squamous cell carcinoma compared to high-grade squamous intraepithelial lesions and normal cervical cells (64), primarily due to its role in catalyzing the hydroxylation of lysine residues in collagen molecules (65, 66). Furthermore, PLOD2 has been identified as a potential therapeutic target for colorectal cancer (67). Notably, the knockdown of PLOD2 markedly reduced migration, invasion, and proliferation in SiHa and HeLa cells, underscoring its potential role in cervical cancer progression. Consequently, PLOD2 presents as a promising therapeutic target for treating this malignancy.

Our study has several limitations. Although the modeling and validation utilized the TCGA database, reliance on publicly available bioinformatics datasets introduced challenges such as data inconsistencies, batch effects, and potential bias, affecting the robustness and generalizability of the NCRS model. Additionally, the absence of independent external samples limited rigorous validation. Future research should use more diverse, multi-center datasets with detailed clinical and experimental data to improve model robustness. Advanced machine learning algorithms and cross-validation techniques could mitigate biases and enhance predictive accuracy. Furthermore, although we identified a relationship between NCRS and the tumor immune microenvironment, as well as responses to immunotherapy, this conclusion was predominantly based on bioinformatics analyses with limited experimental validation. In summary, despite the prognostic model’s potential clinical relevance, additional experimental validation and clinical trials are imperative to enhance its utility.

This study identified eight distinct cell types and their tissue-specific distributions through the analysis of single-cell sequencing data from cervical cancer patients. Notably, EPCs exhibited pronounced proliferative and stemness characteristics. From malignant EPCs, five tumor subpopulations were delineated, with the C1 NNMT+ CAEPCs subpopulation emerging as critical for tumor differentiation, indicating its potential as a therapeutic target. The NNMT CAEPCs Risk Score (NCRS) model effectively predicted patient prognosis; those with elevated NCRS scores experienced poorer outcomes and displayed a stronger association with immune evasion mechanisms. Research on immune-related genes suggests that patients in the high NCRS group may experience enhanced benefits from immunotherapy, especially when combined with CTLA4 and PD1 inhibitors. Moreover, experimental validation highlighted the potential of PLOD2 as a therapeutic target in cervical cancer. This research highlights the importance of early detection and precision therapy by identifying novel treatment targets. Furthermore, it underscores the need to integrate targeted therapies with immunotherapy to enhance patient survival and quality of life. By elucidating interactions within the tumor microenvironment and identifying key cellular subpopulations, this study offers insights that could inform future therapeutic strategies and improve patient outcomes.

## Conclusion

This study analyzed single-cell sequencing data from cervical cancer patients, identifying eight distinct cell types and categorizing malignant EPCs into five unique tumor subpopulations. Among these, C1 NNMT+ CAEPCs were highlighted as a critical therapeutic target for promoting tumor differentiation. The NNMT CAEPCs Risk Score (NCRS) model demonstrated a robust association between poorer patient outcomes and heightened immune evasion, accurately predicting prognosis. Additionally, the validation of PLOD2 as a prognostic gene underscored its therapeutic potential. By emphasizing the importance of integrating immunotherapy with targeted treatment, this research lays the groundwork for early detection, tailored interventions, and improved prognostic outcomes for cervical cancer patients.

## Data Availability

The original contributions presented in the study are included in the article/**Supplementary Material**. Further inquiries can be directed to the corresponding authors.

## Supplementary material

Supplementary Figure 1 Distribution of all cell types in cervical cancer. **(A)** UMAP plots provided a comprehensive overview of the distribution of various cell types in cervical cancer, illustrating the Cell Stemness AUC, G2M Score, tissue origins (H1, H2, T1, T2, T3, T4), and specific phases of the cell cycle (G1, G2M, S). **(B)** A 3D UMAP plot depicted the distribution of different tissue types of cervical cancer cells, including HSIL and tumor samples. **(C)** Each cell type’s proportional proportions across the different tissue origins were displayed in a bar chart. **(D)** The bubble plot displayed the top five marker genes associated with each cell type.

Supplementary Figure 2 InferCNV analysis. **(A)** Through InferCNV analysis, epithelial cells exhibiting significant copy number variation were classified as tumor cells, with gain regions indicated in red and loss regions shown in blue. **(B)** InferCNV analysis illustrated the copy number variation status of different cervical cancer malignant epithelial cell subsets.

Supplementary Figure 3 Prognostic gene analysis. **(A-J)** Ridge plots, complemented by boxplots, illustrated the differential expression levels of NCRS-related genes across the various NCRS groups.
