# Supplementary figures and images for: Single-cell RNA sequencing and immune microenvironment analysis reveal PLOD2-driven malignant transformation in cervical cancer
Source: Front Immunol. 2025 Jan 7;15:1522655. doi: 10.3389/fimmu.2024.1522655 (PMC11747275; doi:10.3389/fimmu.2024.1522655)

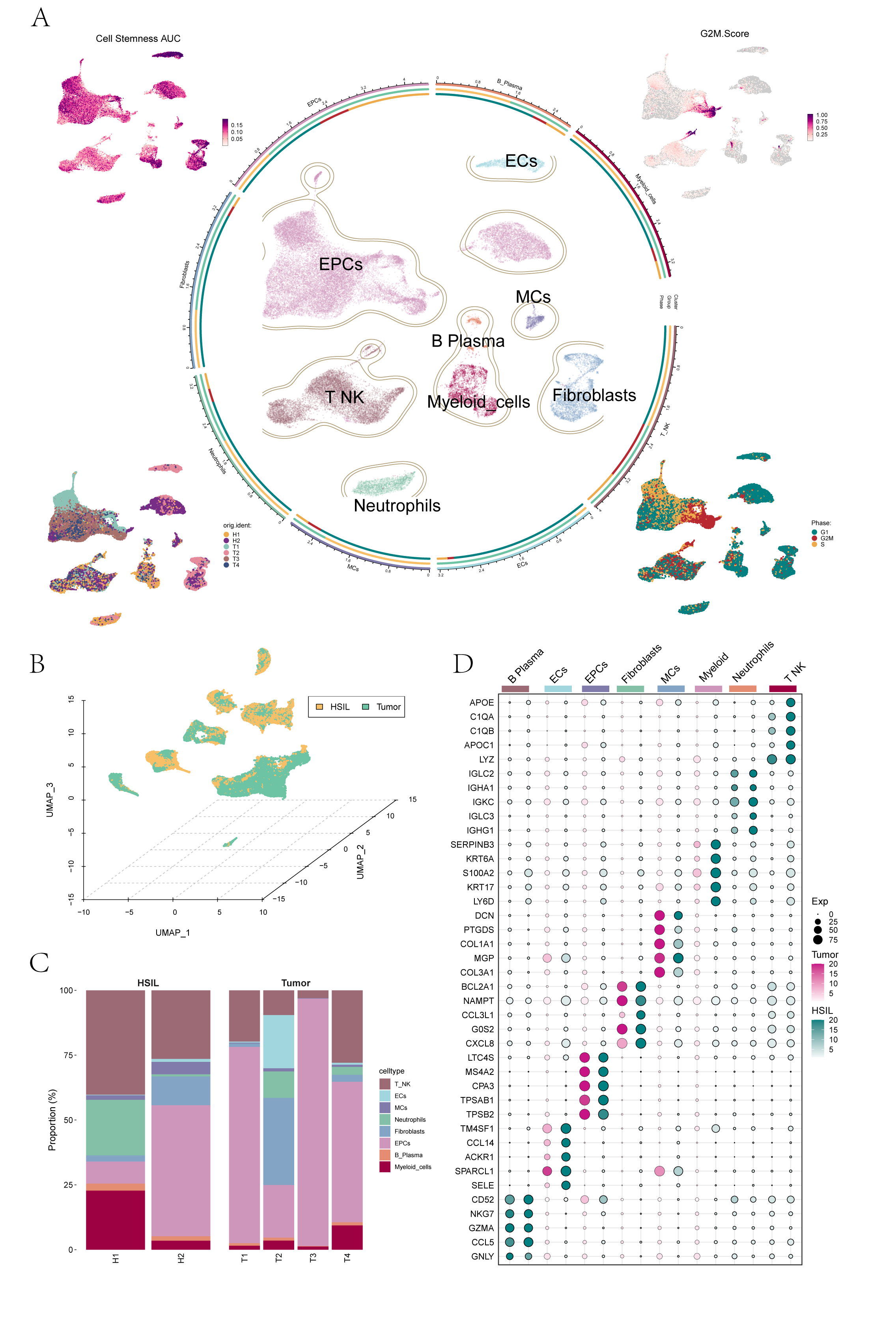

Supplement: Supplementary Figure 1 — Distribution of all cell types in cervical cancer. (A) UMAP plots provided a comprehensive overview of the distribution of various cell types in cervical cancer, illustrating the Cell Stemness AUC, G2M Score, tissue origins (H1, H2, T1, T2, T3, T4), and specific phases of the cell cycle (G1, G2M, S). (B) A 3D UMAP plot depicted the distribution of different tissue types of cervical cancer cells, including HSIL and tumor samples. (C) Each cell type’s proportional proportions across the different tissue origins were displayed in a bar chart. (D) The bubble plot displayed the top five marker genes associated with each cell type. [file Image1.tif]

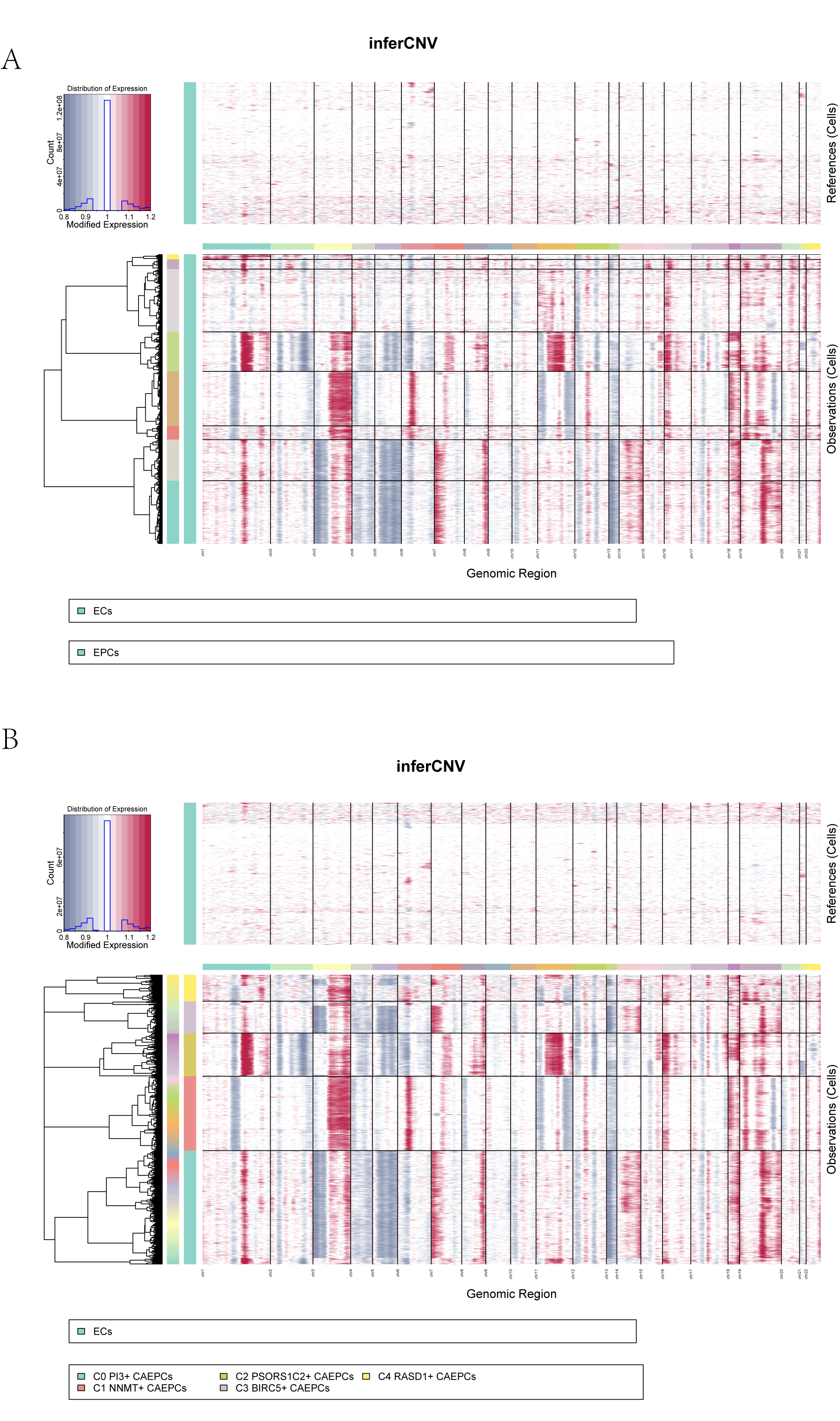

Supplement: Supplementary Figure 2 — InferCNV analysis. (A) Through InferCNV analysis, epithelial cells exhibiting significant copy number variation were classified as tumor cells, with gain regions indicated in red and loss regions shown in blue. (B) InferCNV analysis illustrated the copy number variation status of different cervical cancer malignant epithelial cell subsets. [file Image2.tif]

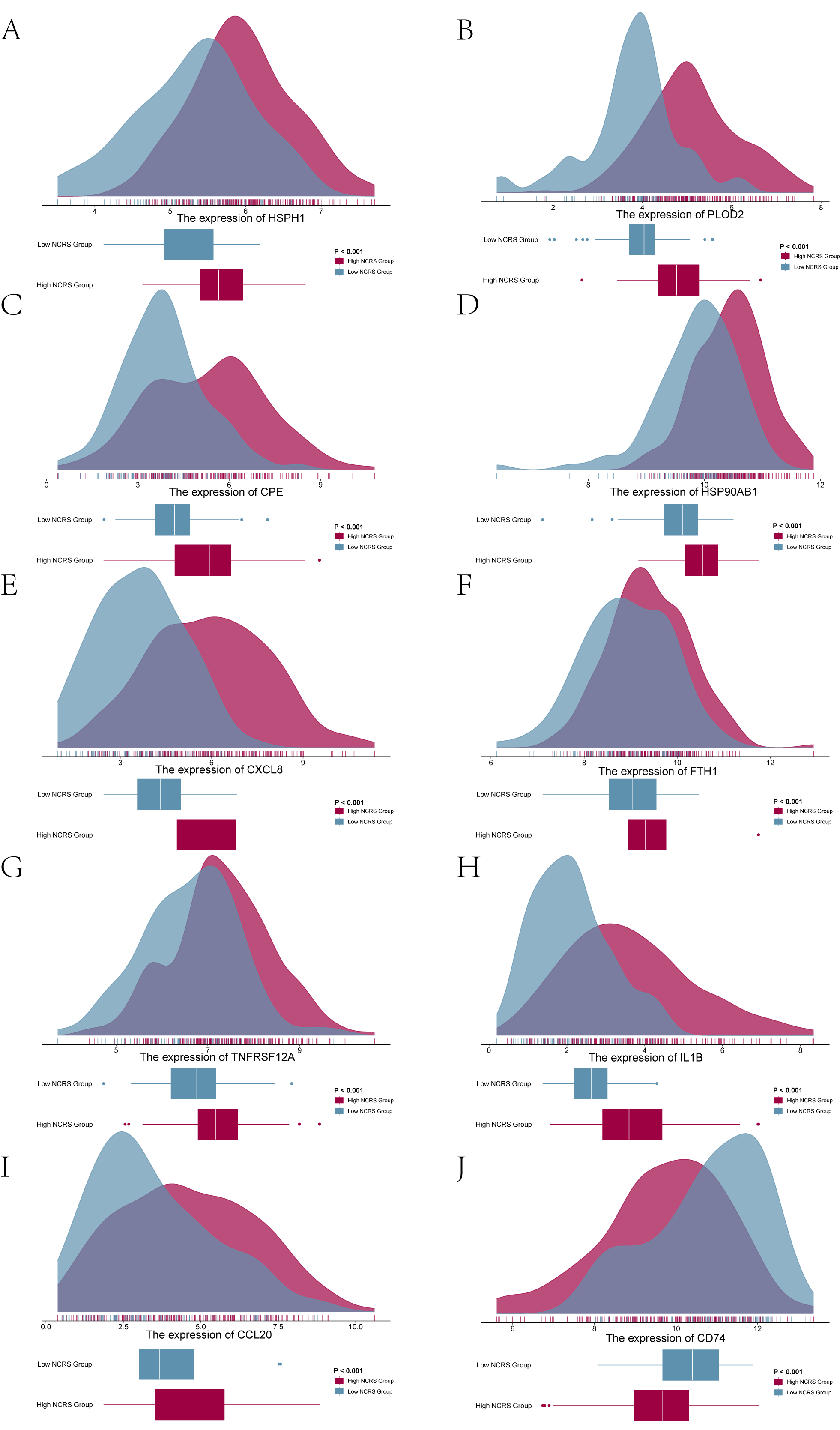

Supplement: Supplementary Figure 3 — Prognostic gene analysis. (A-J) Ridge plots, complemented by boxplots, illustrated the differential expression levels of NCRS-related genes across the various NCRS groups. [file Image3.tif]
